# NLRP3/Caspase-1 Regulate Macrophage Efferocytosis by Modulating ADAM17-Mediated MerTK Cleavage in Liver Ischemia–Reperfusion Injury

**DOI:** 10.34133/research.1122

**Published:** 2026-01-28

**Authors:** Ge Guan, Chaoqun Yu, Longyu Miao, Tao Xiong, Yang Sun, Xiaoshuang Jin, Pengxiang Zhao, Yuerong Lu, Lisheng Wang, Peng Chen, Guohu Di

**Affiliations:** ^1^ Organ Transplantation Center, The Affiliated Hospital of Qingdao University, Qingdao, Shandong, China.; ^2^ Department of Histology and Embryology, Binzhou Medical University, Yantai, Shandong, China.; ^3^ School of Basic Medicine, Qingdao University, Qingdao, Shandong, China.; ^4^ Department of Cardiovascular Surgery, the Affiliated Hospital of Qingdao University, Qingdao University, Qingdao, Shandong, China.; ^5^ Laboratory of Molecular Diagnosis and Regenerative Medicine, Medical Research Center, the Affiliated Hospital of Qingdao University, Qingdao, Shandong, China.; ^6^ Institute of Stem Cell and Regenerative Medicine, School of Basic Medicine, Qingdao University, Qingdao, Shandong, China.

## Abstract

In liver ischemia–reperfusion injury (LIRI), macrophage clearance of apoptotic cells via efferocytosis is crucial to prevent excessive inflammation and tissue damage. Here, we investigate the role of nucleotide-binding oligomerization domain-like receptor protein 3/cysteine-aspartate protease-1 (NLRP3/Caspase-1) signaling in modulating macrophage efferocytosis during LIRI. We observed robust activation of the NLRP3/Caspase-1 pathway during the early phase of LIRI. Genetic ablation of *Nlrp3* or *Caspase-1* substantially reduced LIRI severity. Notably, myeloid-specific *Nlrp3* knockout mice exhibited less severe LIRI compared to hepatocyte-specific *Nlrp3* knockouts, whereas macrophage-specific overexpression of *Caspase-1* exacerbated tissue injury. Mechanistically, NLRP3/Caspase-1 activation enhances a disintegrin and metalloprotease protein-17 (ADAM17)-mediated cleavage of Mer proto-oncogene tyrosine kinase (MerTK), leading to impaired efferocytosis. Pharmacological inhibition of ADAM17 restored macrophage efferocytic capacity and alleviated LIRI. Clinically, elevated serum levels of soluble MerTK (s-Mer) correlated with hepatic injury severity and Caspase-1 activation in patients after partial hepatectomy or liver transplantation. Our findings suggest a potential therapeutic strategy for LIRI prevention and treatment.

## Introduction

Liver ischemia–reperfusion injury (LIRI) is an inevitable pathological process in liver transplantation, hepatectomy, hemorrhagic shock, and various other clinical settings [1]. It serves as the predominant cause of posttransplant liver dysfunction and severe rejection reactions, significantly impacting the postoperative survival rate and quality of life for patients [2,3]. LIRI involves 2 distinct yet interconnected pathological phases: the initial ischemia-induced damage and the subsequent reperfusion-mediated sterile inflammatory response. During the ischemic phase, glycogen depletion, insufficient oxygen delivery, and adenosine triphosphate (ATP) exhaustion result in cellular metabolic disturbances, culminating in hepatocyte death. Upon reperfusion, damaged cells release damage-associated molecular patterns (DAMPs), which activate immune cells and induce the release of proinflammatory cytokines and reactive oxygen species (ROS) [4]. This cascade triggers a robust sterile inflammatory response, further amplifying liver injury [5,6]. Despite its critical clinical relevance, no effective therapeutic strategies have been established to attenuate ischemia–reperfusion (I/R) injury in the liver.

The locally sterile inflammatory response mediated by innate immunity is widely recognized as a critical mechanism in LIRI, and modulating the reperfusion-induced inflammatory response remains a central therapeutic strategy [6–8]. Inflammasomes are multiprotein complexes that play an essential role in the innate immune response by sensing cellular stress and initiating inflammatory signaling through cytokine release. Among these, the nucleotide-binding oligomerization domain-like receptor (NLR) family pyrin domain-containing 3 (NLRP3) inflammasome is currently the most extensively investigated [9,10]. Upon activation, the NLRP3 inflammasome recruits and activates Caspase-1, which processes pro-inflammatory cytokines such as interleukin-1β (IL-1β) and IL-18 into their active forms and promotes their secretion, thereby inducing inflammation and subsequent tissue damage [11,12]. Research has demonstrated that NLRP3 is highly expressed in hepatic macrophages, and the activation of the NLRP3/Caspase-1 pathway in these cells significantly contributes to the pathogenesis of various liver diseases, including LIRI [9,13]. As essential nonparenchymal cells in the liver, macrophages are crucial for maintaining hepatic physiological functions and act as primary immune cells during liver injury [14–17]. The process by which macrophages clear dead or apoptotic cells, termed efferocytosis, plays a pivotal role in resolving inflammation during tissue repair [18]. Prior research has established that aging diminishes the phagocytic capacity of macrophages, thereby exacerbating liver damage [19,20]. Our recent investigation revealed that activation of the NLRP3 inflammasome in macrophages regulates efferocytosis during liver regeneration [21]. Nevertheless, the precise role of the NLRP3/Caspase-1 pathway in modulating efferocytosis within LIRI and the underlying mechanisms remain to be elucidated.

Mer proto-oncogene tyrosine kinase (MerTK) is highly expressed on macrophages and serves as a receptor for recognition during the processes of efferocytosis [22]. It plays a critical role in mediating efferocytosis and resolving inflammation [23]. Under inflammatory conditions, MerTK on the surface of macrophages undergoes proteolytic cleavage, resulting in its functional inactivation. The resultant soluble Mer (s-Mer) competitively inhibits the interaction between MerTK and its ligands, thereby suppressing efferocytosis [24,25]. Studies have demonstrated that MerTK is closely associated with LIRI, nonalcoholic steatohepatitis (NASH), liver fibrosis, and other diseases [26–28]. Further research has identified a disintegrin and metalloprotease protein-17 (ADAM17) as the key enzyme responsible for MerTK cleavage [29]. ADAM17 is a widely distributed transmembrane protein and a member of the zinc-dependent metalloproteinase superfamily. Its primary function involves the proteolytic processing of various precursor membrane proteins, including cytokines, cell adhesion factors, and proteases, which are involved in diverse physiological processes such as inflammation, cell proliferation, and apoptosis [30,31]. However, whether NLRP3/Caspase-1 signaling regulates macrophage efferocytosis by modulating ADAM17 activity remains to be fully elucidated.

In this study, we present evidence demonstrating that the NLRP3/Caspase-1 pathway regulates LIRI via ADAM17/MerTK-mediated macrophage efferocytosis. Moreover, clinical data indicate that serum s-Mer levels are elevated in patients undergoing hepatectomy or liver transplantation and exhibit a significant positive correlation with alanine aminotransferase (ALT) and aspartate aminotransferase (AST) levels. Collectively, our findings provide novel insights and establish a theoretical foundation for the development of future therapeutic strategies.

## Results

### NLRP3 inflammasome activation participates in LIRI

**Fig. 1.**
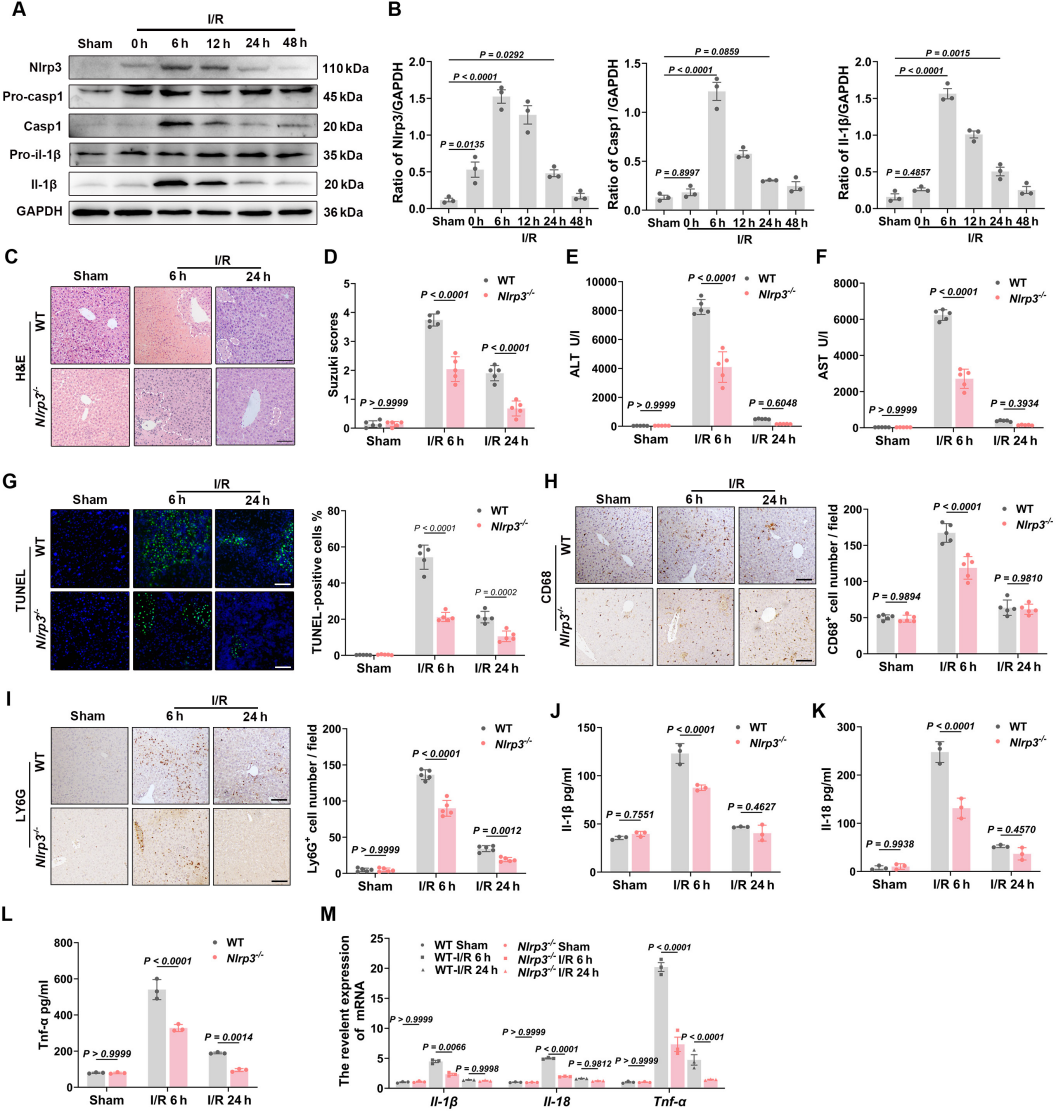
NLRP3/Caspase-1 activation contributes to the pathogenesis of LIRI. (A and B) Western blot analysis and statistical quantification of Nlrp3, pro-Casp1, Casp1, pro-Il-1β, and Il-1β protein levels in WT mouse livers at indicated time points post-reperfusion (*n* = 3 per group). (C)​​ Representative H&E-stained liver sections from WT and *Nlrp3*^*−*/*−*^ mice at specified reperfusion time points (scale bar, 50 μm). (D)​​ Statistical analysis of Suzuki scores in WT and *Nlrp3*^*−*/*−*^ mouse livers at designated time points (*n* = 5 per group). (E and F)​​ Serum ALT and AST levels in WT and *Nlrp3*^*−*/*−*^ mice measured at indicated time points (*n* = 5 per group). (G)​​ Representative TUNEL staining (green) with DAPI (blue) in liver sections and quantitative analysis from WT and Nlrp3^*−*/*−*^ mice (scale bar, 50 μm; *n* = 5 per group). (H) Representative IHC staining of CD68 (macrophage marker) in liver sections and quantitative analysis from WT and *Nlrp3*^*−*/*−*^ mice (scale bar, 50 μm; *n* = 5 per group). (I) Representative IHC staining of Ly6G (neutrophil marker) in liver sections and quantitative analysis from WT and *Nlrp3*^*−*/*−*^ mice (scale bar, 50 μm; *n* = 5 per group). (J to L) ELISA quantification of serum Il-1β, Il-18, and Tnf-α levels in WT and *Nlrp3*^*−*/*−*^ mice at indicated time points (*n* = 3 per group). (M) qRT-PCR analysis of *Il-1β*, *Il-18*, and *Tnf-α* mRNA expression in livers from WT and *Nlrp3*^*−*/*−*^ mice at designated time points (*n* = 3 per group). Data were shown as mean ± SEM.

The LIRI models were established as our previous description, and samples were collected at 0, 6, 12, 24, and 48 h post-reperfusion [32]. Hematoxylin and eosin (H&E) staining and Suzuki scoring demonstrated that liver damage was the most severe at 6 and 12 h after reperfusion (Fig. S1A and B). Concurrently, serum ALT and AST levels were significantly elevated during the same time period (Fig. S1C and D). To explore the role of NLRP3/Caspase-1 in LIRI, quantitative real-time polymerase chain reaction (qRT-PCR) and Western blot analysis revealed that the protein levels of Nlrp3, Casp1, and Il-1β in the mouse liver were markedly increased during the early phase of reperfusion, peaking at 6 h (Fig. S1E and Fig. 1A and B). Enzyme-linked immunosorbent assay (ELISA) results further indicated that the expression levels of the inflammatory cytokines Il-1β, Il-18, and Tnf-α were also significantly up-regulated during the early reperfusion phase (Fig. S1F to H).

### NLRP3 inflammasome deficiency mitigates LIRI

To further investigate the role of NLRP3 in LIRI, *Nlrp3*^*−*/*−*^ mice were utilized and liver tissues were harvested at 6 and 24 h post-reperfusion (Fig. S2A). Histological analysis via H&E staining revealed a significant reduction in liver damage in *Nlrp3*^*−*/*−*^ mice compared to wild-type (WT) controls (Fig. 1C and D). Consistently, serum levels of ALT and AST were markedly decreased in *Nlrp3*^*−*/*−*^ mice (Fig. 1E and F). Moreover, TUNEL (terminal deoxynucleotidyl transferase–mediated deoxyuridine triphosphate nick end labeling) staining demonstrated a substantial reduction in the number of apoptotic cells in the *Nlrp3*^*−*/*−*^ group (Fig. 1G). Inflammatory response serves as the central mechanism in the process of LIRI [33,34]. Immunohistochemical (IHC) staining revealed a markedly reduced infiltration of CD68^+^ macrophages and Ly6G^+^ neutrophils in the livers of *Nlrp3*^*−*/*−*^ mice compared to WT mice (Fig. 1H and I). Additionally, ELISA and qRT-PCR analyses demonstrated that proinflammatory cytokines, such as Il-1β, Il-18, and Tnf-α, exhibited decreased expression at both gene and protein levels in Nlrp3^*−*/*−*^ mice (Fig. 1J to M). Collectively, these findings suggest that NLRP3 activation plays a critical role in LIRI.

### Myeloid-specific deletion of NLRP3 ameliorates LIRI

**Fig. 2.**
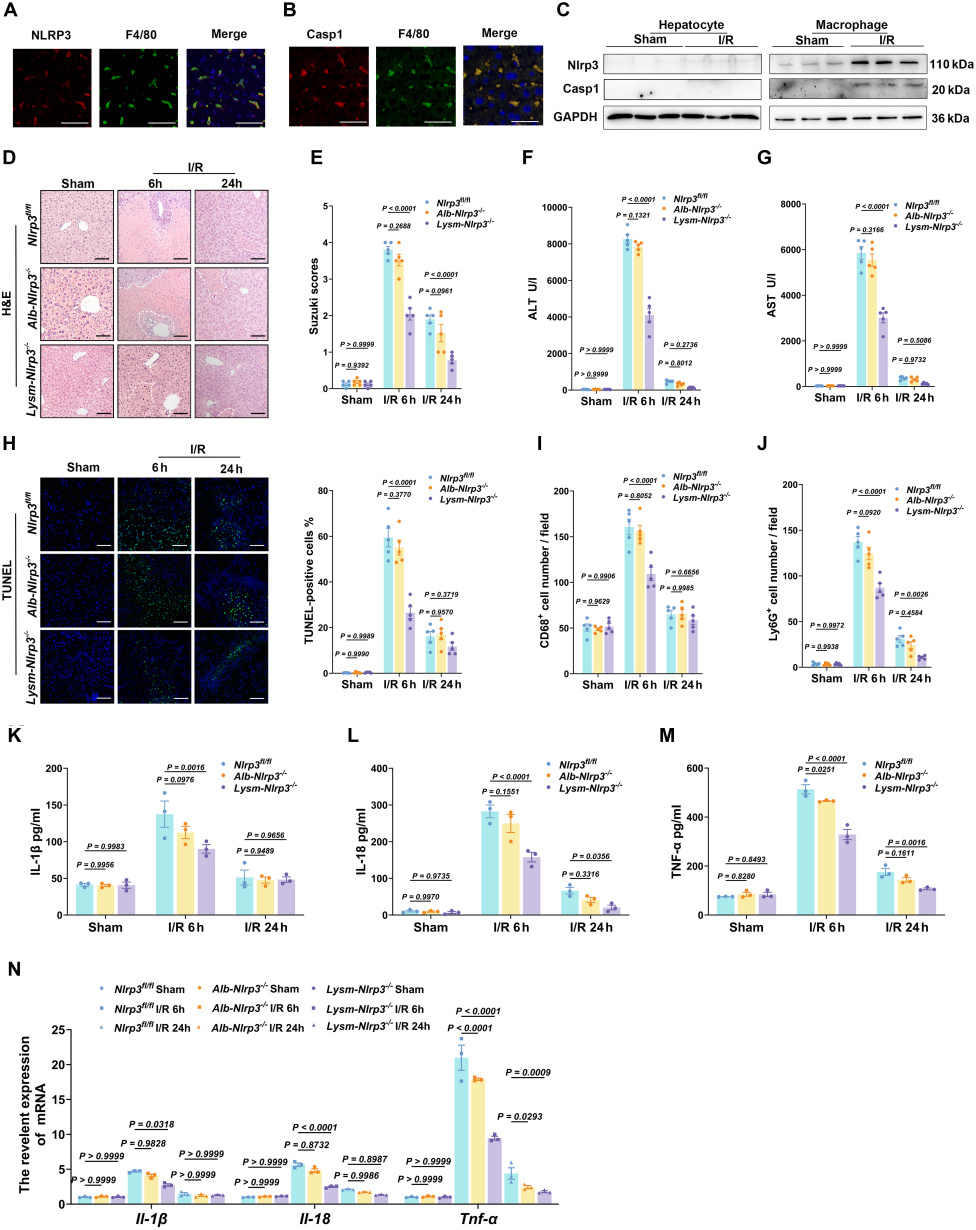
Myeloid-specific Nlrp3 deficiency attenuates LIRI.​​ (A)​​ Representative immunofluorescence costaining of NLRP3 (red), F4/80 (green), and DAPI (blue) in liver sections from WT mice at 6 h post-reperfusion (scale bar, 50 μm). (B)​​ Representative immunofluorescence costaining of Caspase-1 (red), F4/80 (green), and DAPI (blue) in liver sections from WT mice at 6 h post-reperfusion (scale bar, 50 μm). (C)​​ Western blot analysis of Nlrp3 and Caspase-1 protein levels in primary hepatocytes and macrophages from WT mice (*n* = 3 per group). (D)​​ Representative H&E-stained liver sections from *Nlrp3^fl/fl^*, *Alb-Nlrp3*^*−*/*−*^, and *Lysm-Nlrp3*^*−*/*−*^ mice at indicated reperfusion time points (scale bar, 50 μm). (E)​​ Statistical analysis of Suzuki scores in *Nlrp3^fl/fl^*, *Alb-Nlrp3*^*−*/*−*^, and *Lysm-Nlrp3*^*−*/*−*^ mouse livers at designated time points (*n* = 5 per group). (F and G)​​ Serum ALT and AST levels in *Nlrp3^fl/fl^*, *Alb-Nlrp3*^*−*/*−*^, and *Lysm-Nlrp3*^*−*/*−*^ mice measured at indicated time points (*n* = 5 per group). (H) Representative TUNEL staining (green) with DAPI (blue) in liver sections and quantitative analysis from *Nlrp3^fl/fl^*, *Alb-Nlrp3*^*−*/*−*^, and *Lysm-Nlrp3*^*−*/*−*^ mice (scale bar, 50 μm; *n* = 5 per group). (I and J)​​ Statistical analysis of CD68 and Ly6G IHC staining in liver sections from *Nlrp3^fl/fl^*, *Alb-Nlrp3*^*−*/*−*^, and *Lysm-Nlrp3*^*−*/*−*^ mice at designated time points (*n* = 5 per group). (K to M)​​ ELISA quantification of serum IL-1β, IL-18, and TNF-α levels in *Nlrp3^fl/fl^*, *Alb-Nlrp3*^*−*/*−*^, and *Lysm-Nlrp3*^*−*/*−*^ mice at indicated time points (*n* = 3 per group). (N) qRT-PCR analysis of *Il-1β*, *Il-18*, and *Tnf-α* mRNA expression in livers from *Nlrp3^fl/fl^*, *Alb-Nlrp3*^*−*/*−*^, and *Lysm-Nlrp3*^*−*/*−*^ mice at designated time points (*n* = 3 per group). Data were shown as mean ± SEM.

To identify the cellular origin of NLRP3 and Caspase-1, dual immunofluorescence staining was performed on liver tissue from WT mice 6 h post-reperfusion, which showed that NLRP3 or Caspase-1 was predominantly localized to F4/80^+^ macrophages (Fig. 2A and B). To further confirm this, primary hepatocytes and hepatic macrophages were isolated 6 h after reperfusion. Western blot analysis revealed that NLRP3 and Caspase-1 were primarily expressed in macrophages, with significantly increased levels compared to the sham group, while no significant changes were observed in hepatocytes (Fig. 2C).

To explore the association between NLRP3 activation and LIRI in macrophages, we generated macrophage-specific *Nlrp3* knockout mice (*Lysm-Nlrp3*^*−*/*−*^) and hepatocyte-specific Nlrp3 knockout mice (*Alb-Nlrp3*^*−*/*−*^) using the Cre-loxP system (Fig. S2B). Compared to the *Nlrp3^fl/fl^* group, *Lysm-Nlrp3*^*−*/*−*^ mice showed reduced hepatic damage (Fig. 2D and E), lower serum ALT/AST levels at 6 h post-reperfusion (Fig. 2F and G), fewer TUNEL-positive cells (Fig. 2H), diminished immune cell infiltration, and decreased proinflammatory cytokine expression in serum and liver tissues (Fig. 2I to N). In contrast, *Alb-Nlrp3*^*−*/*−*^ mice exhibited no significant changes in any of these results (Fig. 2D to N). These results suggest that NLRP3 depletion in myeloid cells, but not hepatocytes, alleviates LIRI.

### Caspase-1 deficiency mitigates LIRI

**Fig. 3.**
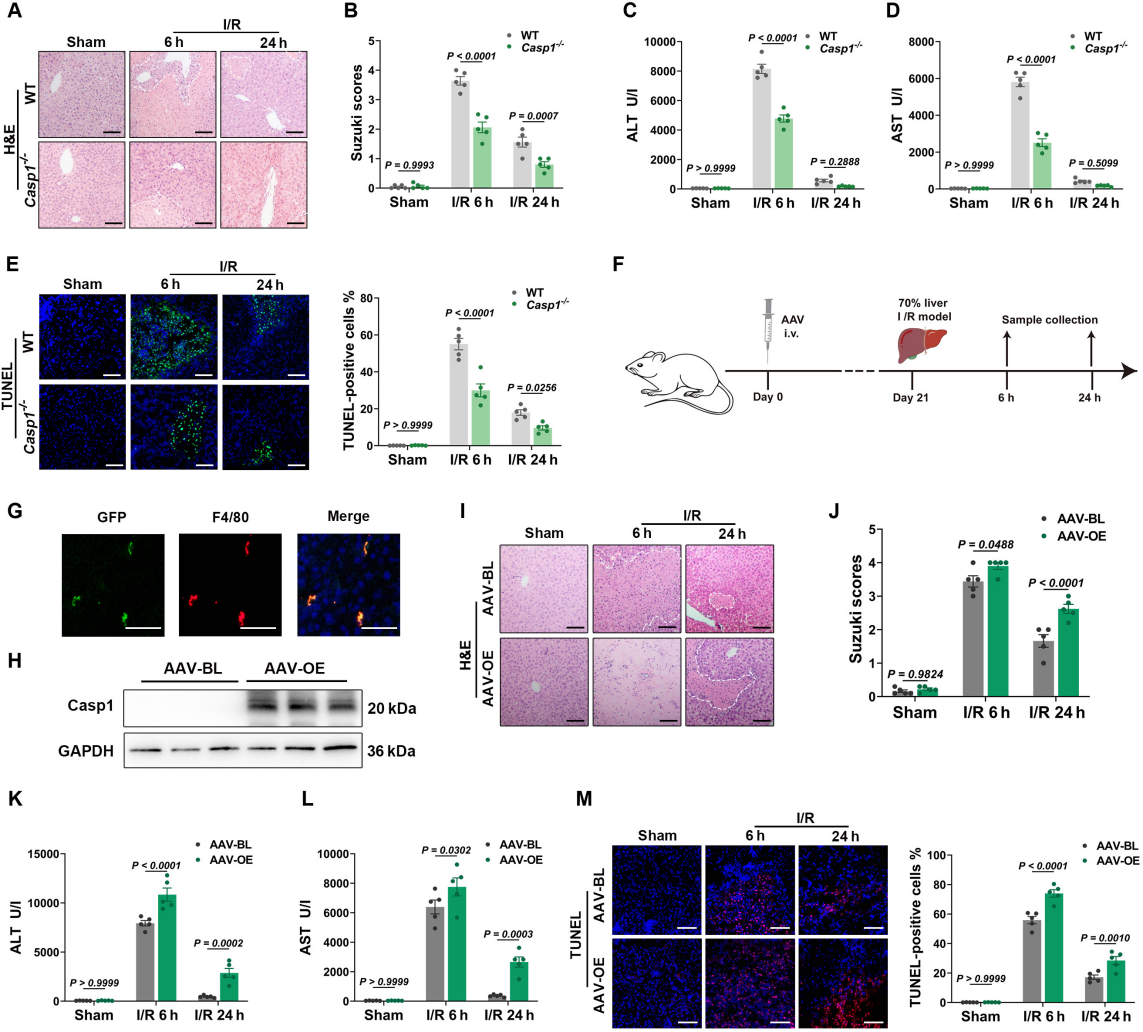
Caspase-1 activation is critical in modulating LIRI. (A)​​ Representative H&E-stained liver sections from WT and *Casp1*^*−*/*−*^ mice at indicated reperfusion time points (scale bar, 50 μm). (B)​​ Statistical analysis of Suzuki scores in livers of WT and *Casp1*^*−*/*−*^ mice at designated time points (*n* = 5 per group). (C and D)​​ Serum ALT and AST levels in WT and *Casp1*^*−*/*−*^ mice measured at indicated time points (*n* = 5 per group). (E)​​ Representative TUNEL staining (green) with DAPI counterstain (blue) in liver sections and quantitative analysis from WT and *Casp1*^*−*/*−*^ mice (scale bar, 50 μm; *n* = 5 per group). (F)​​ Experimental workflow diagram for hepatic macrophage-specific AAV-OE mice. (G)​​ Representative fluorescence images of AAV-OE mouse livers (green, GFP; blue, DAPI; scale bar, 50 μm). (H)​​ Western blot validation of Caspase-1 overexpression in hepatic macrophages of AAV-OE mice (*n* = 3 per group). (I)​​ Representative H&E-stained liver sections from AAV-BL and AAV-OE mice (scale bar, 50 μm). (J)​​ Statistical analysis of Suzuki scores in AAV-BL and AAV-OE mice (*n* = 5 per group). (K and L) Serum ALT and AST levels in AAV-BL and AAV-OE mice at designated time points (*n* = 5 per group). (M)​​ Representative TUNEL staining (red) with DAPI counterstain (blue) in liver sections and quantitative analysis from AAV-BL and AAV-OE mice (scale bar, 50 μm; *n* = 5 per group). Data were shown as mean ± SEM.

The activation of Caspase-1 is critical for the NLRP3 inflammasome function, converting danger signals into an inflammatory cascade by regulating downstream pathways [35–37]. To further elucidate the role of Caspase-1, LIRI model was established using *Casp1*^*−*/*−*^ mice (Fig. S3A). The depletion of *Casp1* mitigated hepatic histopathological damage in mice, as evidenced by H&E staining and Suzuki scoring (Fig. 3A and B). Serum ALT and AST levels were significantly lower (Fig. 3C and D), and TUNEL staining revealed fewer apoptotic cells in *Casp1*^*−*/*−*^ mice (Fig. 3E). Additionally, IHC analysis demonstrated decreased infiltration of CD68^+^ macrophages and Ly6G^+^ neutrophils (Fig. S3B and C). ELISA and qRT-PCR confirmed reduced expression of proinflammatory cytokines (Il-1β, Il-18, Tnf-α) compared to WT controls (Fig. S3D to G). These findings indicate that *Casp1* knockout alleviates LIRI.

### Hepatic macrophage Caspase-1 overexpression exacerbates LIRI

Since Caspase-1 is predominantly expressed in macrophages, as demonstrated above (Fig. 2B and C), we aimed to validate this by establishing liver macrophage-specific *Casp1* overexpression model. To accomplish this, C57BL/6 mice were injected with adeno-associated virus serotype 9 (AAV9) vectors containing the F4/80 promoter-driven enhanced green fluorescent protein (EGFP) (AAV-OE) or empty vector controls (AAV-BL) (Fig. 3F). Immunofluorescence and Western blot analyses confirmed hepatic macrophage-specific *Casp1* overexpression (Fig. 3G and H). Compared with AAV-BL controls, H&E staining revealed significantly exacerbated hepatic injury in AAV-OE mice (Fig. 3I and J), which was further substantiated by markedly elevated serum ALT/AST levels and an increased number of TUNEL^+^ apoptotic cells (Fig. 3K to M). Collectively, these findings indicate that hepatic macrophage-specific *Casp1* overexpression exacerbates LIRI.

### NLRP3/Caspase-1 regulates macrophage efferocytosis

**Fig. 4.**
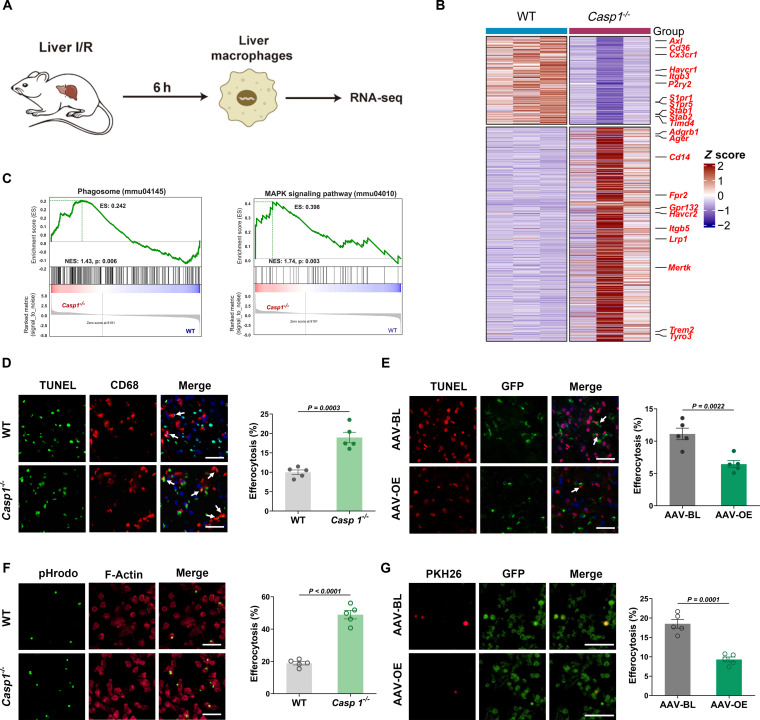
NLRP3/Caspase-1 deficiency enhances macrophage efferocytosis.​​ (A)​​ Schematic workflow of RNA-seq analysis in hepatic macrophages. (B)​​ DEG analysis of hepatic macrophages from WT and *Casp1*^*−*/*−*^ mice at 6 h post-reperfusion. (C) GSEA of hepatic macrophages from WT and Casp1^*−*/*−*^ mice at 6 h post-reperfusion. (D)​​ Representative costaining images [red, CD68 (macrophage marker); green, TUNEL (apoptotic cell marker); blue, DAPI] and quantitative analysis of efferocytosis in liver sections from WT and *Casp1*^*−*/*−*^ mice (scale bar, 50 μm; *n* = 5 per group). (E)​​ Representative costaining images [red, TUNEL; green, GFP (AAV reporter); blue, DAPI] and quantitative analysis of efferocytosis in liver sections from AAV-BL and AAV-OE mice (scale bar, 50 μm; *n* = 5 per group). (F) Representative images and quantitative analysis of F-actin (red)-labeled *Casp1*^*−*/*−*^ BMDMs engulfing pHrodo (green)-labeled apoptotic cells (scale bar, 50 μm; *n* = 5 per group). (G) Representative images and quantitative analysis of GFP-labeled *Casp1*^*−*/*−*^ primary hepatic macrophages engulfing PKH26 (red)-labeled apoptotic cells (scale bar, 50 μm; *n* = 5 per group). Data were shown as mean ± SEM.

To elucidate the underlying mechanisms of LIRI mediated by NLRP3/Caspase-1 activation, hepatic macrophages were isolated from mice at 6 h post-modeling for transcriptome profiling (Fig. 4A). RNA sequencing (RNA-seq) analysis demonstrated a significant up-regulation of efferocytosis-related genes in hepatic macrophages derived from *Casp1*^*−*/*−*^ mice, with these genes being enriched in pathways associated with efferocytosis (Fig. 4B). Furthermore, RNA-seq analysis revealed that the transcriptional profiles of *Nlrp3*^*−*/*−*^ mice exhibited high similarity to those of *Casp1*^*−*/*−*^ mice (Fig. S4A to C). Differential expression analysis (DEG) identified up-regulated efferocytosis-related genes in hepatic macrophages from *Casp1*^*−*/*−*^ mice. Gene set enrichment analysis (GSEA) further confirmed the significant activation of the phagosome pathway [Kyoto Encyclopedia of Genes and Genomes (KEGG) ID: 04145] and the mitogen-activated protein kinase (MAPK) signaling pathway (KEGG ID: 04010) in macrophages (Fig. 4C). Collectively, these findings suggest that NLRP3/Caspase-1 may contribute to LIRI pathogenesis by modulating macrophage efferocytosis.

Prompt and efficient efferocytosis by macrophages facilitates the resolution of tissue inflammation [38,39]. In situ immunofluorescence staining revealed that the proportion of TUNEL^+^CD68^+^ cells in liver tissues was significantly higher in *Nlrp3*^*−*/*−*^ mice and *Casp1*^*−*/*−*^ mice compared to WT mice (Fig. 4D and Fig. S4D). Conversely, the proportion of TUNEL^+^CD68^+^ cells in the livers of AAV-OE mice was markedly reduced compared to the AAV-BL group (Fig. 4E). To further investigate the role of NLRP3/Caspase-1 in macrophage-mediated efferocytosis, coculture experiments demonstrated that genetic ablation of Nlrp3 or Casp1 significantly enhanced in vitro efferocytosis compared to controls (Fig. 4F and Fig. S4E), whereas overexpression of Casp1 substantially impaired this phagocytic process (Fig. 4G). Collectively, these findings suggest that the NLRP3/Caspase-1 pathway may play a critical role in regulating macrophage efferocytosis.

### NLRP3/Caspase-1 activation-mediated MerTK cleavage suppresses macrophage efferocytosis​

**Fig. 5.**
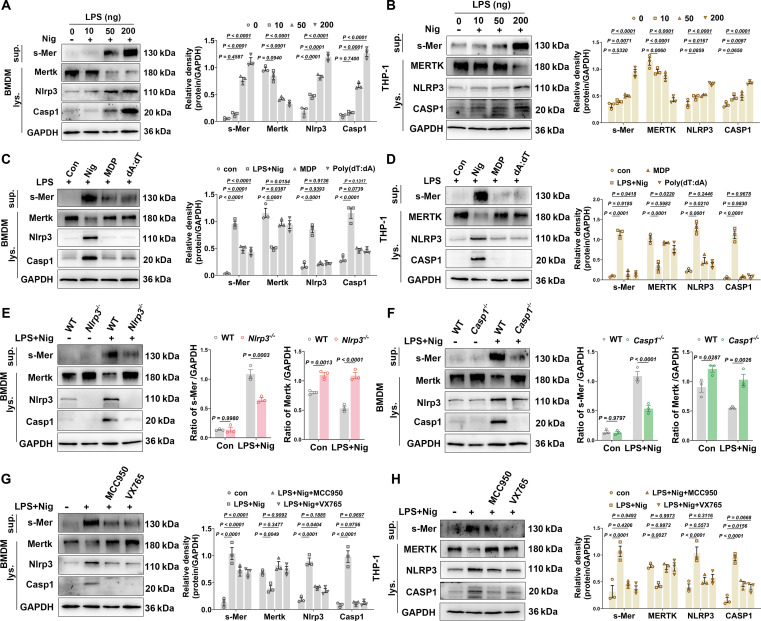
NLRP3/Caspase-1 regulates macrophage MerTK cleavage.​​ (A) Western blot analysis and statistical quantification of s-Mer, Mertk, Nlrp3, and Casp1 protein levels in WT BMDM treated with 0, 10, 50, or 200 ng/ml LPS for 2 h followed by 10 μM nigericin for 1 h (*n* = 3 per group). (B)​​ Western blot analysis and statistical quantification of s-Mer, MERTK, NLRP3, and CASP1 in THP-1-derived macrophages treated as in (​​A)​​ (*n* = 3 per group). (C)​​ Western blot analysis and statistical quantification of s-Mer, Mertk, Nlrp3, and Casp1 in WT BMDM treated with NLRP3/Caspase-1-specific activators (LPS + nigericin: 200 ng/ml LPS for 1.5 h + 10 μM nigericin for 0.5 h), NLRP1-specific activator (MDP: 500 ng/ml for 16 h), or AIM2-specific activator [poly(dA:dT)/LyoVec] (*n* = 3 per group). (D)​​ Western blot analysis and statistical quantification of s-Mer, MERTK, NLRP3, and CASP1 in THP-1-derived macrophages treated as in (​​C)​​ (*n* = 3 per group). (E) Western blot analysis and statistical quantification of s-Mer, Mertk, Nlrp3, and Casp1 in WT and *Nlrp3*^*−*/*−*^ BMDM treated with 200 ng/ml LPS for 2 h followed by 10 μM nigericin for 1 h (*n* = 3 per group). (F)​​ Western blot analysis and statistical quantification of s-Mer, Mertk, Nlrp3, and Casp1 in WT and *Casp1*^*−*/*−*^ BMDM treated as in (​​E)​​ (*n* = 3 per group). (G)​​ Western blot analysis and statistical quantification of s-Mer, Mertk, Nlrp3, and Casp1 in WT BMDM pretreated with inhibitors (NLRP3 inhibitor: 10 μM MCC950; Caspase-1 inhibitor: 55 μM VX765, 0.5 h) followed by 200 ng/ml LPS (2 h) and 10 μM nigericin (1 h) (*n* = 3 per group). (H)​​ Western blot analysis and statistical quantification of s-Mer, MERTK, NLRP3, and CASP1 in THP-1-derived macrophages treated as in (​​G)​​ (*n* = 3 per group). Data were shown as mean ± SEM.

Our prior investigations have demonstrated that MerTK-mediated efferocytosis plays a pivotal role in LIRI and liver regeneration [21,32]. To explore the regulatory role of NLRP3/Caspase-1 on MerTK cleavage, we exposed bone marrow-derived macrophages (BMDMs) or THP-1-derived macrophages to lipopolysaccharide (LPS) at various concentrations, followed by nigericin stimulation. Our results revealed that LPS (10 to 200 ng/ml) dose-dependently activated NLRP3/Caspase-1 signaling in macrophages while concurrently promoting MerTK cleavage (Fig. 5A and B). Notably, this regulatory effect was absent when macrophages were stimulated with the AIM2 inflammasome agonist poly(deoxyadenosine-deoxythymidine) [poly(dA:dT)] or the NLRP1b inflammasome agonist muramyl dipeptide (MDP) (Fig. 5C and D). Additionally, s-Mer levels were significantly diminished in the culture supernatants of *Nlrp3*^*−*/*−*^ or *Casp1*^*−*/*−*^ deficient BMDMs compared to WT controls (Fig. 5E and F). Furthermore, flow cytometry analysis confirmed an increased proportion of MerTK-positive cells in the *Nlrp3*^*−*/*−*^ or *Casp1*^*−*/*−*^ groups (Fig. S5). Consistently, a hypoxia/reoxygenation model applied to BMDMs demonstrated that genetic deletion of either *Nlrp3* or *Casp1*significantly attenuated the hypoxia/reoxygenation-induced increase in s-Mer levels in the culture supernatant compared to WT controls (Fig. S6). Similarly, pretreatment of macrophages with the NLRP3-specific inhibitor MCC950 or the Caspase-1-specific inhibitor VX765 effectively suppressed MerTK cleavage (Fig. 5G and H).

### Caspase-1-mediated ADAM17 maturation and activation drives MerTK cleavage​

**Fig. 6.**
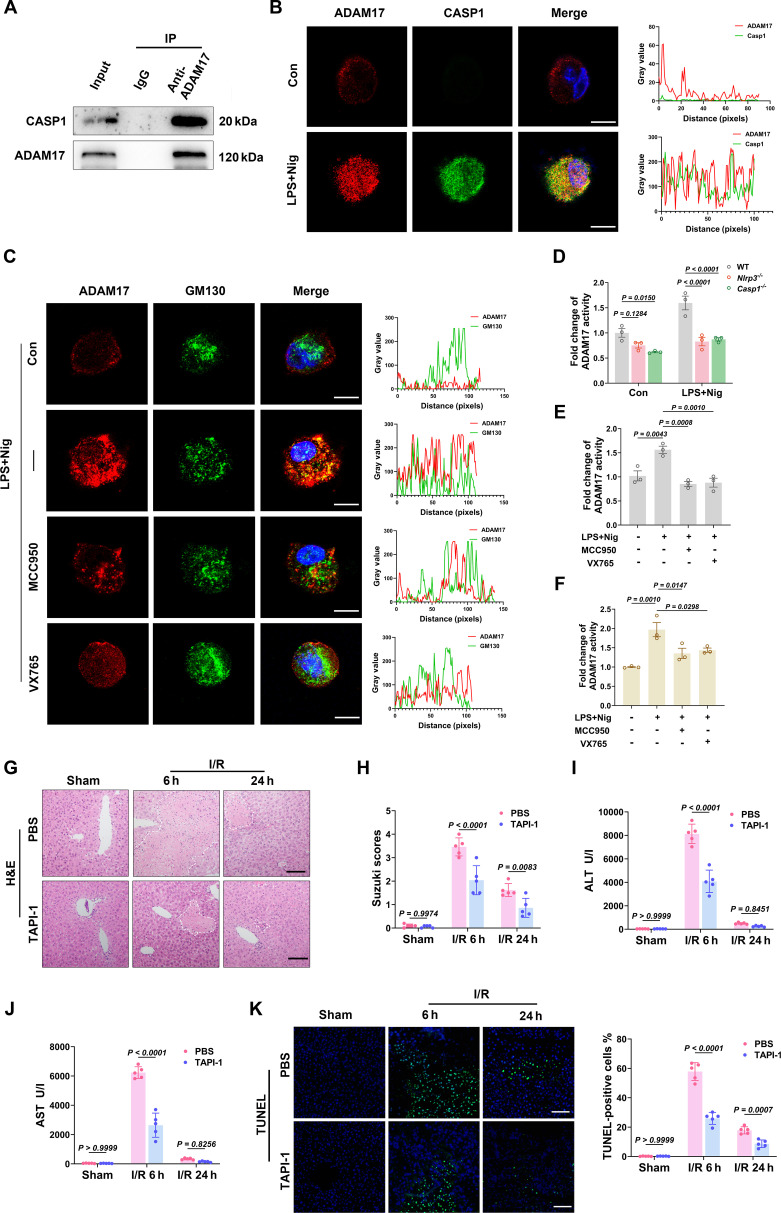
Caspase-1 regulates ADAM17 maturation and activity. (A) Co-IP analysis of CASP1 and ADAM17 interaction in THP-1-derived macrophages treated with 200 ng/ml LPS for 2 h followed by 10 μM nigericin for 1 h. (B)​​ Confocal microscopy images (green, CASP1; red, ADAM17) and quantitative analysis of colocalization in THP-1-derived macrophages treated as in (​​A)​​. (C)​​ Confocal microscopy images (red, ADAM17; green, Golgi marker GM130) and quantitative analysis of ADAM17 localization in THP-1-derived macrophages under indicated conditions. (D)​​ ADAM17 enzymatic activity in BMDM from WT, *Nlrp3*^*−*/*−*^, and *Casp1*^*−*/*−*^ mice (*n* = 3 per group). (E) ADAM17 activity in WT BMDM under indicated conditions (*n* = 3 per group). (F) ADAM17 activity in THP-1-derived macrophages under indicated conditions (*n* = 3 per group). (G)​​ Representative H&E-stained liver sections from PBS-treated and TAPI-1 (ADAM17 inhibitor)-treated mice at designated reperfusion time points (scale bar, 50 μm). (H)​​ Statistical analysis of Suzuki scores in PBS-treated and TAPI-1-treated mice (*n* = 5 per group). (I and J)​​ Serum ALT and AST levels in PBS-treated and TAPI-1-treated mice measured at indicated time points (*n* = 5 per group). (K) Representative TUNEL staining (green) with DAPI counterstain (blue) in liver sections and quantitative analysis from PBS-treated and TAPI-1-treated mice (scale bar, 50 μm; *n* = 5 per group). Data were shown as mean ± SEM.

As MerTK cleavage is predominantly dependent on ADAM17, we first investigated whether the NLRP3/Caspase-1 pathway regulates ADAM17 at the transcriptional or translational level. qRT-PCR and Western blot analyses of liver tissues from WT, *Nlrp3*^*−*/*−*^, and *Casp1*^*−*/*−*^ mice revealed that neither the mRNA (Fig. S7A and B) nor the protein (Fig. S7C and D) expression levels of ADAM17 were significantly altered by genetic deletion of *Nlrp3* or *Casp1*. This indicates that the observed regulatory effects are independent of ADAM17 expression. However, and critically, measurement of soluble Mer (s-Mer) levels in the serum of these mice showed a marked reduction in both *Nlrp3*^*−*/*−*^ and *Casp1*^*−*/*−*^ mice compared to WT controls after I/R injury (Fig. S7E and F). Therefore, we focused subsequent investigations on the posttranslational regulation of ADAM17 activity. Coimmunoprecipitation (Co-IP) assays showed strong interaction between ADAM17 and Caspase-1 in LPS + Nig-stimulated THP-1 macrophages (Fig. 6A). Moreover, confocal microscopy revealed significantly up-regulated ADAM17 and Caspase-1 expression in the LPS + Nig group compared to controls, with substantial colocalization (Fig. 6B). To confirm if Caspase-1 promotes ADAM17 trafficking to the Golgi apparatus for maturation, we performed dual immunofluorescence labeling of ADAM17 and GM130, followed by confocal analysis. Quantitative analysis showed increased ADAM17-GM130 overlap in LPS + Nig-stimulated cells compared to controls, which was attenuated by NLRP3 or Caspase-1 inhibitors (Fig. 6C). Moreover, ADAM17 activity assays demonstrated that LPS + Nig stimulation induced a significant elevation of ADAM17 enzymatic activity, which could be reversed by genetic ablation of *Nlrp3* or *Casp1*, as well as pharmacological inhibitors (Fig. 6D to F). Rescue experiments demonstrated that pharmacological activation of ADAM17 restored s-Mer levels in the culture supernatants of *Nlrp3*^*−*/*−*^ and *Casp1*^*−*/*−*^ BMDMs, while its inhibition by TAPI-1 suppressed s-Mer production in WT BMDMs, confirming that NLRP3/Caspase-1 regulates MerTK cleavage primarily by modulating ADAM17 activity (Fig. S8). Furthermore, in vivo studies in LIRI mouse models showed that TAPI-1 treatment alleviated histopathological damage (Fig. 6G and H), reduced serum ALT/AST levels (Fig. 6I and J), and decreased TUNEL-positive cell counts (Fig. 6K). These results suggest that NLRP3/Caspase-1-mediated ADAM17 signaling is critical for LIRI progression.

### s-Mer affects postoperative recovery of TL and PHx patients

**Fig. 7.**
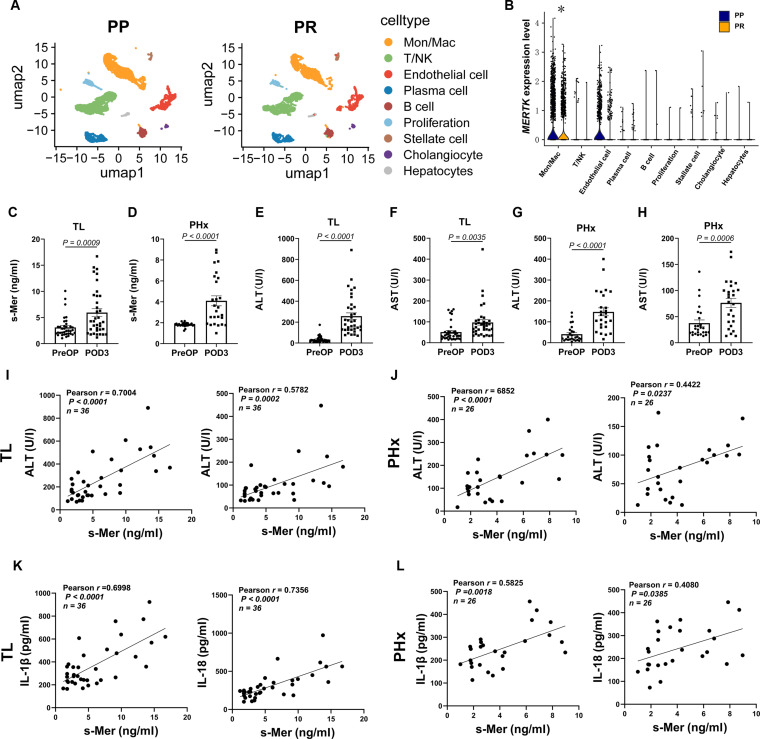
s-Mer levels influence postoperative liver injury in patients undergoing TL and PHx.​​ (A) Differences in cellular composition between PreOP and POD3 samples in TL patients, visualized via t-distributed stochastic neighbor embedding (t-SNE) plots. (B)​​ Violin plots showing MERTK expression across differential cell subsets in PreOP and POD3 groups (TL patients). (C) ELISA quantification of serum s-Mer levels in TL patients (*n* = 36). (D)​​ ELISA quantification of serum s-Mer levels in PHx patients (*n* = 26). (E)​​ ELISA quantification of ALT levels in TL patients (*n* = 36). (F)​​ ELISA quantification of AST levels in TL patients (*n* = 36). (G)​​ ELISA quantification of ALT levels in PHx patients (*n* = 26). (H) ELISA quantification of AST levels in PHx patients (*n* = 26). (I)​​ Pearson correlation between serum s-Mer and ALT/AST levels in TL patients at POD3 (*n* = 36). (J)​​ Pearson correlation between serum s-Mer and ALT/AST levels in PHx patients at POD3 (*n* = 26). (K) Pearson correlation between serum s-Mer and IL-1β/IL-18 levels in TL patients at POD3 (*n* = 36). (L)​​ Pearson correlation between serum s-Mer and IL-1β/IL-18 levels in PHx patients at POD3 (*n* = 26). Data were shown as mean ± SEM.

Single-cell RNA sequencing (scRNA-seq) data (GSE171539) from the Gene Expression Omnibus (GEO) database were analyzed, including one pre-procurement (PP) and one post-reperfusion (PR) liver sample. Unsupervised clustering identified 13 clusters annotated as 9 cellular populations based on marker gene expression (Fig. 7A and Fig. S9A to D). Wilcoxon rank-sum tests showed significantly reduced MERTK expression in the PR group compared to the PP group (Fig. S9E and F). Subgroup analysis revealed the most substantial MERTK down-regulation in monocyte/macrophage populations in the PR cohort (Fig. 7B). To investigate the correlation between s-Mer and postoperative recovery in liver transplantation (TL) and partial hepatectomy (PHx) patients, individuals undergoing single-step hepatectomy and liver transplantation were enrolled (details summarized in Tables S1 and S2). Consistent with single-cell analysis findings, circulating s-Mer levels significantly increased on POD3 in both TL and PHx patients (Fig. 7C and D). This was accompanied by elevated serum ALT and AST levels (Fig. 7E to H). To ensure that the correlation between s-Mer levels and liver injury markers was not influenced by confounding factors, we analyzed the potential impact of cold ischemia time on these variables. Analysis revealed no significant correlation between cold ischemia time and s-Mer levels, ALT, or AST, indicating that these associations are not influenced by cold ischemia time (Fig. S10). Further correlation analysis showed strong positive associations between s-Mer levels and hepatocellular injury markers (ALT/AST) (Fig. 7I and J). Moreover, in TL patients, elevated s-Mer levels were further correlated with impaired coagulation function, as indicated by positive associations with the international normalized ratio (INR) and prolonged prothrombin time (PT), and with a longer duration of hospital stay (Fig. S11). Additionally, s-Mer levels correlated significantly with Caspase-1 activation markers, including IL-1β and IL-18 (Fig. 7K and L).

## Discussion

**Fig. 8.**
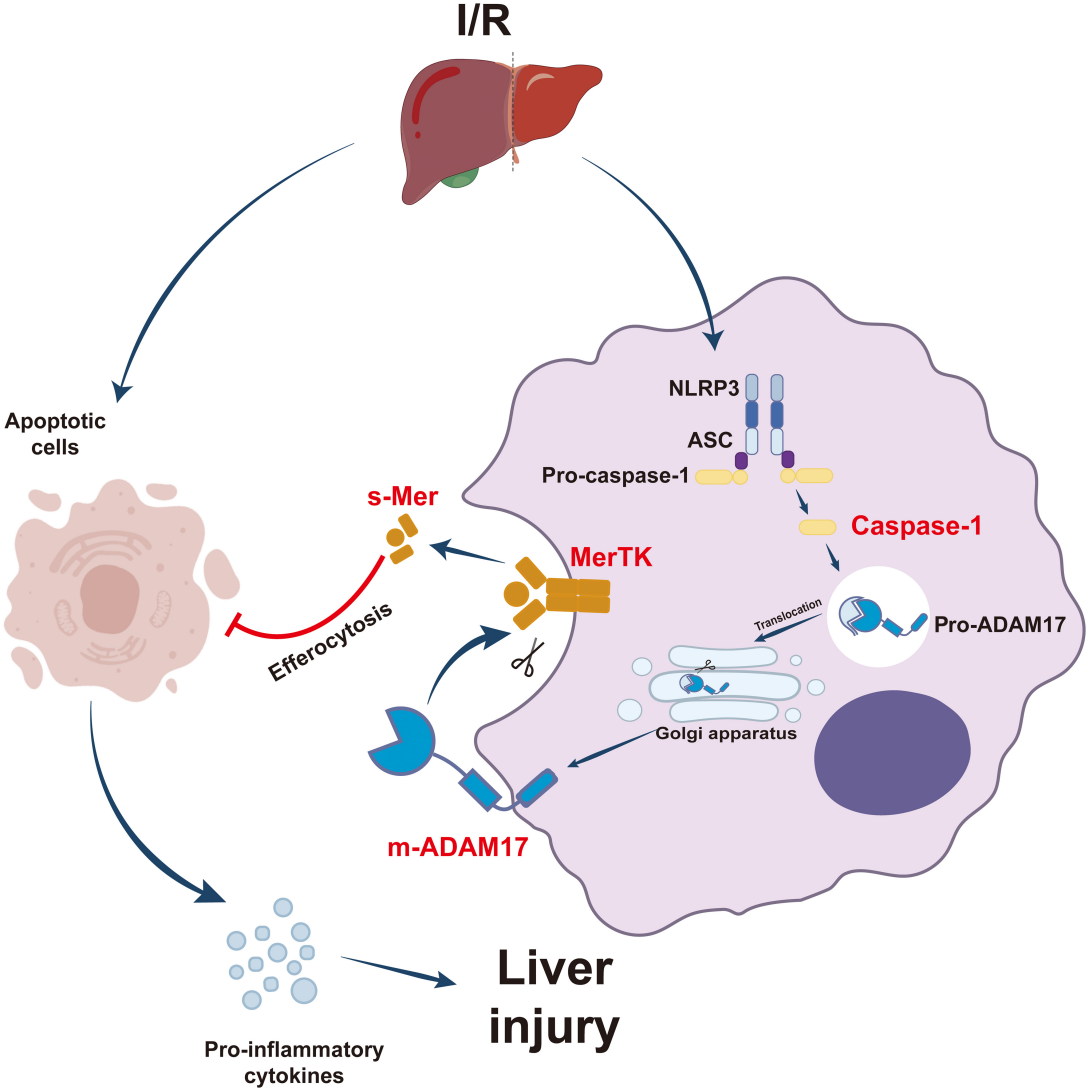
The schematic diagram shows the potential role and mechanisms of NLRP3/Caspase-1 in LIRI.

In this study, we systematically investigated the functions and mechanisms of NLRP3/Caspase-1 in the pathogenesis of LIRI. Our results demonstrate that the activation of NLRP3/Caspase-1 in macrophages is critical for LIRI progression. We also found that impaired ADAM17-MerTK-mediated efferocytosis drives LIRI due to NLRP3/Caspase-1 activation. Additionally, our data reveal a correlation between circulating s-Mer levels and postoperative outcomes in patients undergoing liver surgery, offering clinical insights for targeting LIRI complications (Fig. 8).

During the pathological progression of LIRI, macrophages play dual roles as initiators of proinflammatory cascades and critical effectors in tissue repair [6,40,41]. The NLRP3 inflammasome, which is highly expressed in macrophages, serves as a key mediator in various acute and chronic liver diseases [13,42,43]. Previous studies have demonstrated that the NLRP3 inflammasome and Caspase-1 are activated during LIRI, and genetic deletion of *Nlrp3* or *Casp1* confers protective effects against LIRI [13,44]. Consistent with these prior reports, our findings indicate that *Nlrp3* or *Casp1* deficiency mitigates LIRI. Notably, we further identified that NLRP3/Caspase-1 is specifically activated in macrophages, but not in hepatocytes. Furthermore, myeloid-specific *Nlrp3* deficiency alleviates LIRI, while macrophage-specific Caspase-1 overexpression exacerbates injury, suggesting that NLRP3/Caspase-1 mediates LIRI pathogenesis by modulating macrophage functional states. Indeed, during ischemia/reperfusion, timely macrophage-mediated clearance of apoptotic cells is essential for minimizing tissue damage [45,46]. Intriguingly, RNA-seq analysis revealed that *Nlrp3* or *Casp1* deletion leads to transcriptomic reprogramming, with significant changes observed in genes and signaling pathways associated with efferocytosis. Moreover, both in situ and in vitro efferocytic assays confirmed that *Nlrp3* or *Casp1* ablation markedly enhances the clearance efficiency of apoptotic cells.

MerTK, a highly expressed phagocytic receptor on macrophages, undergoes proteolytic cleavage under inflammatory conditions to generate s-Mer, which inhibits efferocytosis [47,48]. Recent studies have demonstrated that cleavage-resistant MerTK (*Mertk^CR^*) mice exhibit robust protective effects in models of inflammation and cardiovascular disease [28,49], whereas loss-of-function mutations in MerTK result in severe outcomes, including impaired macrophage phagocytosis and polarization [50,51]. Furthermore, the inactivation of MerTK induced by proteolytic cleavage impairs post-ischemic repair following myocardial I/R injury, promotes plaque necrosis and defective resolution in atherosclerosis, and exacerbates tissue inflammation by restricting the biosynthesis of proresolving mediators, thereby accelerating disease progression [28,49,52]. In our previous study, we demonstrated that NLRP3 inflammasome activation regulates MerTK-mediated efferocytosis in macrophages, contributing to delayed liver regeneration. Notably, primary hepatic macrophages derived from *Nlrp3*^*−*/*−*^ and *Casp1*^*−*/*−*^ mice after LIRI exhibited markedly up-regulated MerTK expression, suggesting an inverse regulatory relationship between NLRP3/Caspase-1 signaling and efferocytosis. Additionally, activation of the NLRP3/Caspase-1 pathway in BMDMs and THP-1-induced macrophages led to increased levels of s-Mer in culture supernatants, which can be reversed by genetic or pharmacological inhibition of NLRP3 or Caspase-1. Other inflammasomes like AIM2 and NLRP1 did not affect s-Mer expression, indicating that MerTK cleavage is specifically dependent on NLRP3/Caspase-1 activation.

ADAM17-mediated cleavage is dynamically regulated by protease activity, metabolic reprogramming, and inflammatory signaling [31,53]. However, its upstream regulators exhibit significant heterogeneity across different pathological contexts. Accumulating evidence indicates that ROS, inflammatory cytokines [e.g., tumor necrosis factor-α (TNF-α) and IL-1β], and other factors modulate ADAM17 activity [54,55]. Notably, ROS, TNF-α, and IL-1β converge through interconnected networks to regulate NLRP3 inflammasome activity. Zhou and colleagues [20,56] demonstrated that aging exacerbates LIRI by promoting NLRP3 activation in macrophages and enhancing ROS production, which induces ADAM17-mediated MerTK cleavage, thereby inhibiting efferocytosis and worsening LIRI. Although these studies did not directly establish a link between NLRP3 and ADAM17-MerTK-mediated efferocytosis, they suggest an interaction between NLRP3 signaling and efferocytosis dysfunction. Moreover, activated Caspase-1, a key effector of the NLRP3 inflammasome, has been increasingly recognized for its roles beyond cytokine maturation, including the regulation of enzyme activities and signaling pathways via noncanonical substrate cleavage [57]. The sheddase activity of ADAM17 depends on its dynamic trafficking from the endoplasmic reticulum to the Golgi apparatus and precursor processing [58]. Our results reveal that NLRP3/Caspase-1 activation promotes ADAM17-Golgi translocation, confirming that Caspase-1 accelerates ADAM17 maturation and enzymatic activation by facilitating its Golgi translocation. Furthermore, treatment with the ADAM17-specific inhibitor TAPI-1 attenuates LIRI in mice, highlighting the pathological significance of the ADAM17-MerTK-mediated macrophage efferocytosis in LIRI.

The advancement of noninvasive serum biomarkers constitutes a pivotal frontier in modern medicine, with accumulating evidence highlighting the expanding significance of s-Mer in pathological diagnostics. Prior studies have indicated s-Mer as a promising candidate for predicting sepsis-related organ damage and hepatitis B virus (HBV)-associated acute-on-chronic liver failure [59,60]. In this study, our findings underscore the biological relevance of s-Mer by demonstrating markedly elevated serum s-Mer levels in patients undergoing liver transplantation and partial hepatectomy. To substantiate this, single-cell transcriptomic analysis revealed a substantial decrease in MERTK^+^ monocytes/macrophages following liver transplantation. Moreover, s-Mer exhibited robust temporal correlations with hepatocellular injury markers (ALT/AST) and Caspase-1 activation markers (IL-1β/IL-18 levels). Collectively, these findings proposed circulating s-Mer as a novel noninvasive biomarker for diagnosing and monitoring postoperative outcomes in LT/PHx patients.

Several limitations of this study warrant consideration and highlight avenues for future investigation. First, although our findings indicate that NLRP3/Caspase-1 activation promotes ADAM17-mediated MerTK cleavage, the detailed molecular mechanism by which Caspase-1 regulates ADAM17 maturation and intracellular trafficking remains incompletely defined. Further application of proximity-dependent biotin labeling approaches may help identify potential interacting proteins or posttranslational modifications involved in this regulatory axis. Second, although our study has focused on macrophage efferocytosis, the use of a global Casp1 knockout model means that potential contributions from other cell types (e.g., neutrophils or dendritic cells) within the NLRP3/Caspase-1–ADAM17–MerTK signaling network cannot be excluded. Employing cell type-specific (such as ADAM17) knockout or overexpression models of key pathway components will be essential to dissect their cell-autonomous functions in LIRI. Finally, despite identifying circulating s-Mer as a candidate biomarker and demonstrating the efficacy of pharmacological inhibition in mice, further validation in larger, independent patient cohorts will be crucial for evaluating the translational potential of targeting this pathway.

In conclusion, this study highlights the critical role of macrophage NLRP3/Caspase-1 signaling in the pathogenesis of LIRI. Inhibition of the NLRP3/Caspase-1 pathway may mitigate LIRI by suppressing ADAM17 activity, thereby reducing MerTK cleavage mediated by ADAM17 and promoting macrophage endocytosis. Moreover, our findings establish a direct correlation between elevated levels of s-Mer and clinical outcomes in patients, providing robust support for targeting the macrophage NLRP3/Caspase-1–ADAM17–MerTK axis as a therapeutic strategy for managing LIRI.

## Materials and Methods

### Patient samples​

A total of 52 subjects were enrolled in this study, all from Qingdao University Affiliated Hospital. Among them, 26 patients underwent partial hepatectomy (PHx) and 36 patients underwent liver transplantation (LT). Blood samples were routinely collected from patients at 24 h before surgery (PreOP) and 3 days after surgery (POD3). All serum samples were stored at −80 °C.

### Animals

Male C57BL/6 mice (6 to 8 weeks old) were purchased from Beijing Huafukang Biotechnology Co. Ltd. *Nlrp3* knockout mice (*Nlrp3*^*−*/*−*^) and *Caspase-1* knockout mice (*Casp1*^*−*/*−*^) were purchased from Shanghai Nanfang Model Biotechnology Co. Ltd. *Nlrp3-Floxp* and *Alb-Cre* mice were purchased from GemPharmatech Co. Ltd. *Lysm-Cre* mice were purchased from Viewsolid Biotech Co. Ltd. Macrophage-specific *Nlrp3* knockout gene mice (*Lysm-Nlrp3*^*−*/*−*^) and liver cell-specific *Nlrp3* knockout gene mice (*Alb-Nlrp3*^*−*/*−*^) were obtained by crossbreeding mice with *Lysm-Cre* mice or *Alb-Cre* mice, respectively [21,61,62]. All strains of mice were backcrossed for at least 10 generations on a pure C57BL/6 background. All mice were housed in the specific pathogen-free (SPF) animal room of Qingdao University, with a light/dark cycle of 12 h (8:30 AM to 8:30 PM), temperature maintained at 23 ± 2 °C, relative humidity maintained at 60% ± 5%, and free access to water and food.

### Murine model of LIRI and treatment

Male mice were subjected to 70% LIRI as our previous description [32]. Briefly, the mice were fasted for 12 h and anesthetized via inhalation of 2% isoflurane gas. Noninvasive microvascular clips were applied to occlude the blood supply to the left and middle lobes of the liver for 60 min, followed by clip removal to allow reperfusion. For ADAM17 inhibition, TAPI-1 (TargetMol, T6009) was intraperitoneally administered at a dose of 10 mg/kg 1 h prior to LIRI induction. The Sham group underwent the same procedure without vascular occlusion. Mice were euthanized at each designated time point post-reperfusion, and liver and serum samples were immediately collected for subsequent analysis.

### Cell culture and treatment

Primary BMDMs were isolated and cultured as our previous description [*32*]. THP-1 cells were maintained in RPMI 1640 medium supplemented with 10% fetal bovine serum, 0.05 mM β-mercaptoethanol, and 1% penicillin/streptomycin solution. To induce differentiation, THP-1 cells were treated with 100 ng/ml phorbol 12-myristate 13-acetate (PMA) for 24 h. Following which, the culture medium was replaced with fresh growth medium devoid of PMA, and the cells were allowed to rest for an additional 24 h prior to experimental use. All cultures were incubated at 37 °C under 5% CO_2_ in a humidified atmosphere.

For inflammasome stimulation**,** THP-1 cells differentiated into macrophages or BMDMs were serum-starved in RPMI or Dulbecco’s modified Eagle’s medium (DMEM) high-glucose basal medium for 4 h. The cells were then pretreated or not with inhibitors (NLRP3 inhibitor: 10 μM MCC950; Caspase-1 inhibitor: 55 μM VX765) for 0.5 h, followed by treatment with 10, 50, or 200 ng/ml LPS for 2 h. Subsequently, the medium was replaced with Opti-MEM medium containing various inflammatory stimuli at the following concentrations and times: nigericin (10 μM for 1 h, Solarbio), MDP (500 ng/ml for 16 h, TargetMol), and poly(dA:dT)/LyoVec (2 μg/ml for 16 h, Invivogen). After stimulation, the supernatant of the culture medium and cell lysate were collected separately for experiments.

### Biochemical analysis and ELISA

Serum levels of ALT, AST, IL-1β, IL-18, and TNF-α in mice, as well as ALT, AST, s-Mer, IL-1β, and IL-18 in humans, were quantified using ELISA kits (Jianglai Bio) following the manufacturer’s instructions. Absorbance was measured at 450 nm using a SpectraMax microplate reader (Molecular Devices, LLC).

### Western blot analysis

As previously described, Western blot analysis was performed on murine liver tissue or cell lysates. Briefly, protein samples were separated by sodium dodecyl sulfate–polyacrylamide gel electrophoresis (SDS-PAGE) using 7.5%, 10%, or 12.5% gels (Yamay Bio) and transferred to polyvinylidene difluoride (PVDF) membranes via NcmBlot Rapid Transfer Buffer (NCM Biotech). Membranes were blocked with 5% nonfat milk dissolved in tris-buffered saline/Tween 20 (TBST: 150 mM NaCl, 50 mM tris–HCl, pH 7.5, 0.2% Tween 20) for 90 min at room temperature, followed by overnight incubation at 4 °C with primary antibodies (see Table S3 for details). After washing the membranes 3 times with TBST, they were incubated with the corresponding horseradish peroxidase (HRP)-conjugated antibodies for 1 h at room temperature. The membrane was washed 3 times with TBST. Finally, protein bands were imaged using an automated chemiluminescence image analysis system (Tanon), and expression levels of all target proteins were normalized to glyceraldehyde-3-phosphate dehydrogenase (GAPDH) using ImageJ software.

### Histopathology and TUNEL assay

Histological sectioning was performed as previously described [21]. In short, the left lateral lobe of mouse liver tissue was fixed in a 4% paraformaldehyde solution and subsequently embedded in paraffin or optimal cutting temperature (OCT) compound. The paraffin-embedded samples were sectioned at a thickness of 5 μm, while the frozen samples were sectioned at 7 μm. For pathological evaluation of liver tissue, H&E staining was applied to the paraffin sections. For the TUNEL assay, frozen sections were stained using the TUNEL reagent kit (Yeasen) according to the manufacturer’s protocol to detect apoptotic cells. Positive cells were visualized under a fluorescence microscope.

### IHC staining

Paraffin-embedded tissue sections were immersed in citrate buffer and subjected to high-pressure antigen retrieval using a pressure cooker for 10 min. Nonspecific tissue staining was minimized by incubating the sections with 3% hydrogen peroxide at room temperature for 10 min. IHC staining was performed by incubating the sections with primary antibodies (see Table S3 for details), followed by visualization using 3,3′-diaminobenzidine (DAB) chromogenic substrate (Servicebio).

### Immunofluorescence staining

Cryosections (8 μm) of tissue were prepared and subjected to fluorescence microscopy. Dual immunofluorescence staining was performed to evaluate immune cell infiltration using F4/80 antibody and NLRP3 antibody (see Table S3 for details). Nuclear counterstaining was conducted with 4′,6-diamidino-2-phenylindole (DAPI), and positive cells were visualized under a fluorescence microscope.

### Efferocytosis assay

For the in vitro efferocytosis assay, BMDMs were seeded into 48-well plates and pretreated with or without inhibitors for 0.5 h. Apoptotic neutrophils labeled with pHrodo or PKH26 dye were cocultured with BMDMs or hepatic macrophages at a ratio of 5:1 (ACs/M φ, where M φ represents BMDMs) for 2 h. Subsequently, non-adherent cells were removed by washing with phosphate-buffered saline (PBS). BMDMs were further stained with Actin Tracker Red-555 (Beyotime). Fluorescence microscopy was used to capture images for identifying the uptake of labeled apoptotic cells. The phagocytic efficiency was assessed as the percentage of pHrodo green-positive cells among all Actin Tracker Red-555-positive cells or the percentage of PKH26 red-positive cells among all GFP-positive cells.

### Quantitative real-time PCR

Total RNA was extracted from liver tissue or cells using FastPure Cell/Tissue Total RNA Isolation kit (Vazyme), and complementary DNA was synthesized with a PrimeScript First Strand cDNA synthesis kit (TaKaRa) according to the manufacturer’s protocol. qRT-PCR was performed using Synergy Brands (SYBR) Premix Ex Taq (Vazyme) according to standard operating procedures on the Bio Rad CFX96 real-time system (Bio Rad), and the housekeeping gene GAPDH was used as an internal control. The relative expression of each mRNA was calculated using the 2^−ΔΔCq^ method.

### ADAM17 enzyme activity assay

ADAM17 enzyme activity was determined using a SensoLyte 520 TACE Activity Assay Kit (AnaSpec), according to the manufacturer’s protocol. Fluorescence of the cleavage product was measured in a fluorescence microplate reader (EnVision) at an excitation of 490 nm and emission of 520 nm.

### scRNA-seq analysis

scRNA-seq data (GSE171539) were downloaded from the GEO database, including raw FASTQ files from one PP and one PR liver sample. Data processing was performed using Cell Ranger (v8.0.1) with the GRCh38 human reference genome, followed by Seurat (v5.1.0) analysis. After quality control (cell count ≥ 10, gene count ≥ 200, mitochondrial genes ≤ 20%, hemoglobin genes ≤ 5%), doublets were removed using scDblFinder. Data were normalized, and variable features (2,000 genes) were identified. Cell cycle scoring was conducted using canonical S/G2/M phase gene sets. Integration of samples was optimized via canonical correlation analysis (CCA). Dimensionality reduction was performed using principal components analysis (PCA) (30 components) and Uniform Manifold Approximation and Projection (UMAP) for visualization. Cell annotation leveraged CellMarker 2.0 markers and Seurat’s AddModuleScore function. Differential expressions (Wilcoxon test, log_2_FC > 0.25, adjusted *P* < 0.05) and enrichment analyses (clusterProfiler) were performed. *P* < 0.05 was considered to indicate a statistically significant difference.

### Statistical analysis

In this study, all experiments were conducted at least 3 times. The results were expressed as mean ± SEM and statistically analyzed using Prism 10.0 software (GraphPad, San Diego, CA, USA). The unpaired Student’s *t* test is used to determine differences between 2 groups, while one-way analysis of variance (ANOVA) is used to determine differences between multiple groups. *P* < 0.05 was considered to indicate a statistically significant difference.

### Study approval

All animal studies in this project followed the ARRIVE guidelines and were approved by the Ethics Committee of Qingdao University School of Medicine (no.: QDU-AEC-2022309). Written informed consent was obtained from all surgical patients. Sample collection complied with the 1975 Helsinki Declaration and was approved by the Ethics Committee of Qingdao University School of Medicine (no.: QDU-HEC-2024254). Written informed consent was obtained from all study participants. Detailed patient information is shown in Tables S1 and S2.

## Data Availability

All data needed to evaluate the conclusions in the paper are present in the paper and/or the Supplementary Materials. Any materials described in the manuscript can be provided by G.D. pending scientific review and a completed material transfer agreement. Request for the materials should be submitted to the corresponding authors G.D. (diguohu@qdu.edu.cn) or C.Y. (ychaoqunu@126.com) or P.C. (chenpeng@qdu.edu.cn).
