# Supplementary material for: NLRP3/Caspase-1 Regulate Macrophage Efferocytosis by Modulating ADAM17-Mediated MerTK Cleavage in Liver Ischemia–Reperfusion Injury
Source: Research (Wash D C). 2026 Jan 28;9:1122. doi: 10.34133/research.1122 (PMC12848890; doi:10.34133/research.1122)
Supplement: Supplementary 1 — Figs. S1 to S11 Tables S1 to S3 [file research.1122.f1.pdf]

Supplementary Materials for

NLRP3/Caspase-1 orchestrate macrophage efferocytosis via modulating ADAM17mediated MerTK cleavage in liver ischemia-reperfusion injury

Ge Guan#, Chaoqun Yu#, Longyu Miao# *et al.*

*Correspondence author: Guohu Di. Email: diguohu@qdu.edu.cn
**Correspondence author: Chaoqun Yu. Email: ychaoqunu@126.com
***Correspondence author: Peng Chen. Email: chenpeng@qdu.edu.cn

This PDF file includes:

Figs. S1 to S11
Tables S1 to S3

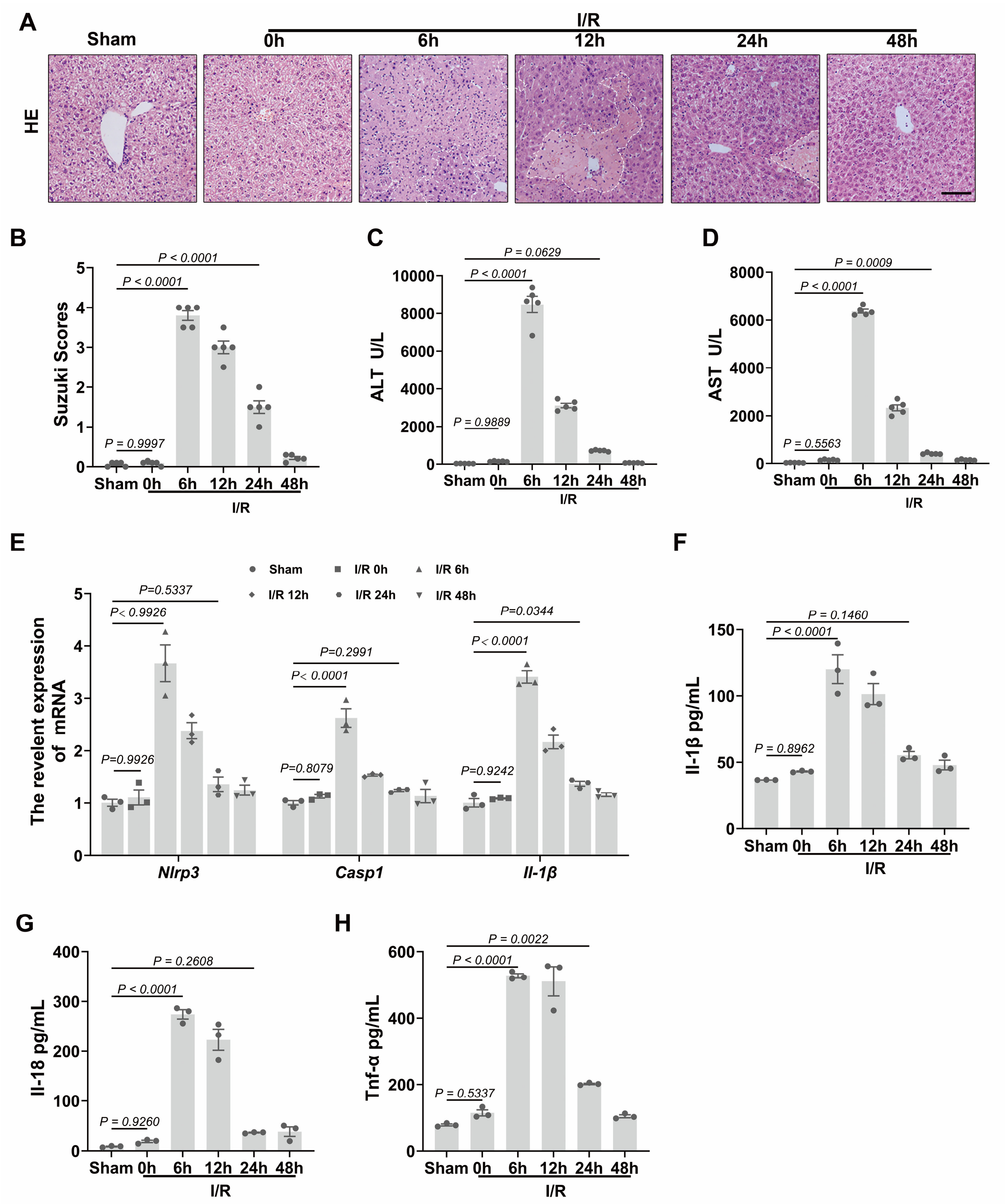

Fig. S1. NLRP3/Caspase-1 Activation in LIRI.(**A**) Representative H&E-stained liver sections from WT mice at specified time points post-reperfusion (scale bar = 50 μm). (**B**) Quantitative analysis of Suzuki Scores in WT mouse livers at specified time points post-reperfusion (n=5 per group). (**C** and **D**) Serum ALT and AST levels in WT mice measured at specified time points post-reperfusion (n=5 per group). (**E**) qRT-PCR analysis of *Nlrp3*, *Caspase-1*, and *Il-1β* mRNA expression in WT mouse livers at specified time points post-reperfusion (n=3 per group). (**F** to

H) ELISA quantification of serum IL-1β, IL-18, and TNF-α secretion in WT mice at specified time points post-reperfusion (n=3 per group). Data was shown as Mean ± SEM.

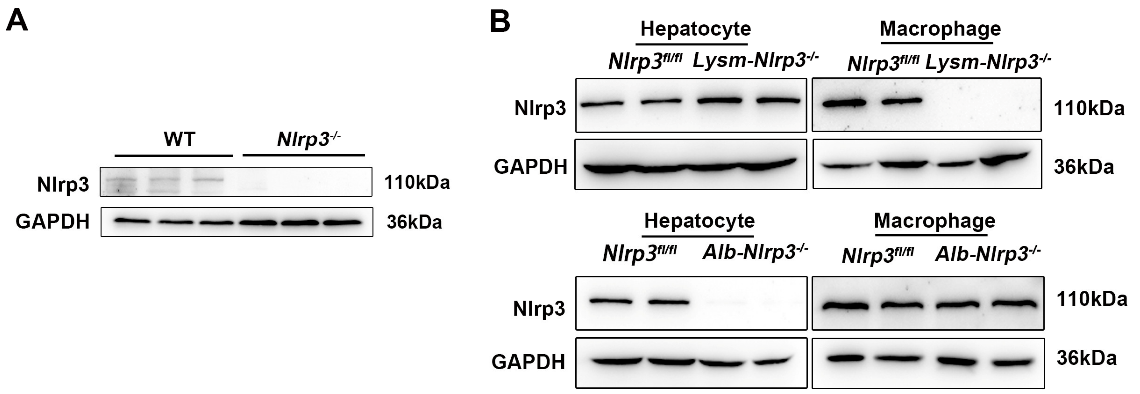

Fig. S2. Validation of *Nlrp3* Knockout Efficiency. (**A**) Western Blot analysis of Nlrp3 protein expression in livers from *Nlrp3*$^{-/-}$ mice (n=3 per group). (**B**) Western Blot validation of Nlrp3 knockout efficiency in hepatocytes or hepatic macrophages isolated from *Nlrp3* conditional knockout mice (n=4 per group).

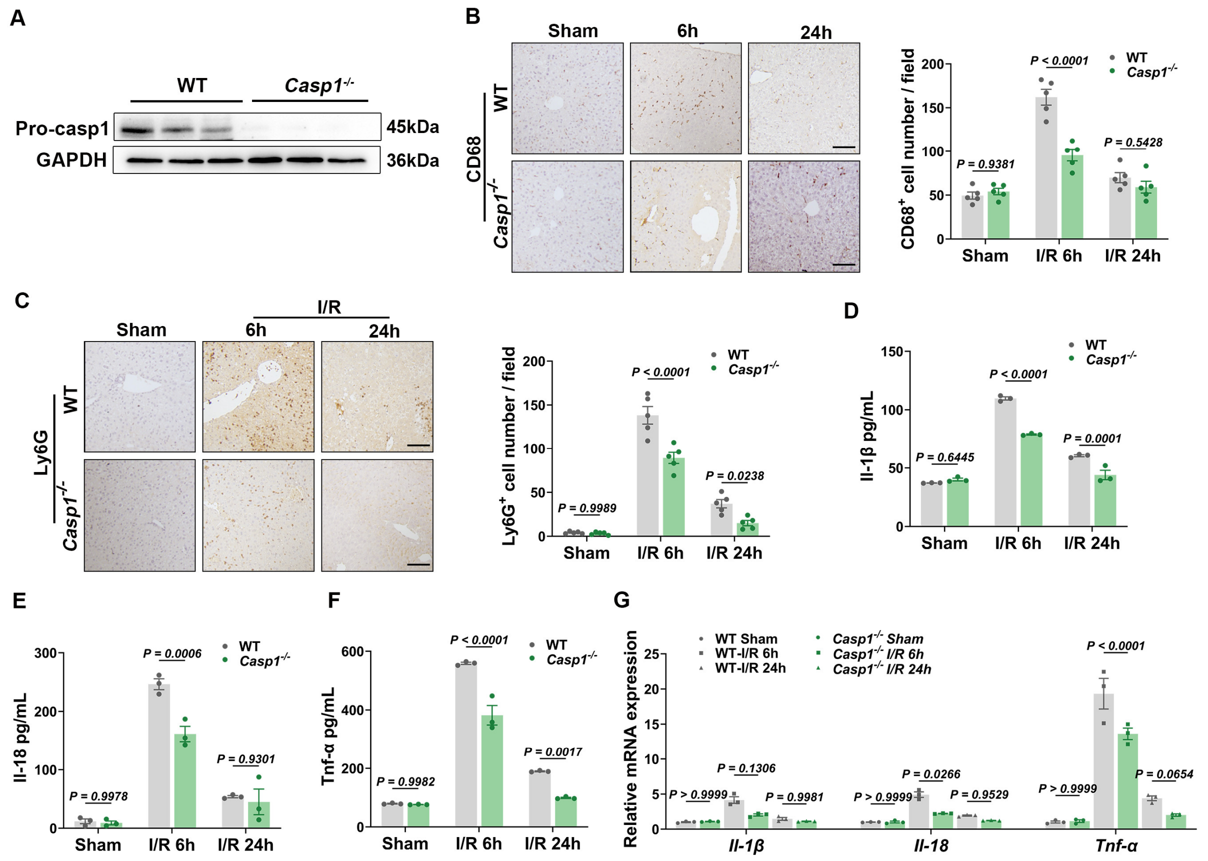

Fig. S3. Caspase-1 Deficiency Attenuates Inflammatory Response in LIRI. (**A**) Western Blot validation of *Casp1* knockout efficiency in livers from *Casp1*$^{-/-}$ mice (n=3 per group). (**B**) Representative immunohistochemical staining (scale bar = 50 μm) and quantitative analysis of CD68^{+} macrophages in liver sections from WT and *Casp1*$^{-/-}$ mice at specified time points post-reperfusion (n=5 per group). (**C**) Representative immunohistochemical staining (scale bar = 50 μm) and quantitative analysis of Ly6G^{+} neutrophils in liver sections from WT and *Casp1*$^{-/-}$ mice at specified time points post-reperfusion (n=5 per group). (**D** to **F**) ELISA quantification of serum Il-1β, Il-18, and Tnf-α levels in WT and *Casp1*$^{-/-}$ mice at specified time points post-reperfusion (n=3 per group). (**G**) qRT-PCR analysis of *Il-1β*, *Il-18*, and *Tnf-α* mRNA expression in livers from WT and *Casp1*$^{-/-}$ mice at specified time points post-reperfusion (n=3 per group). Data was shown as Mean ± SEM.

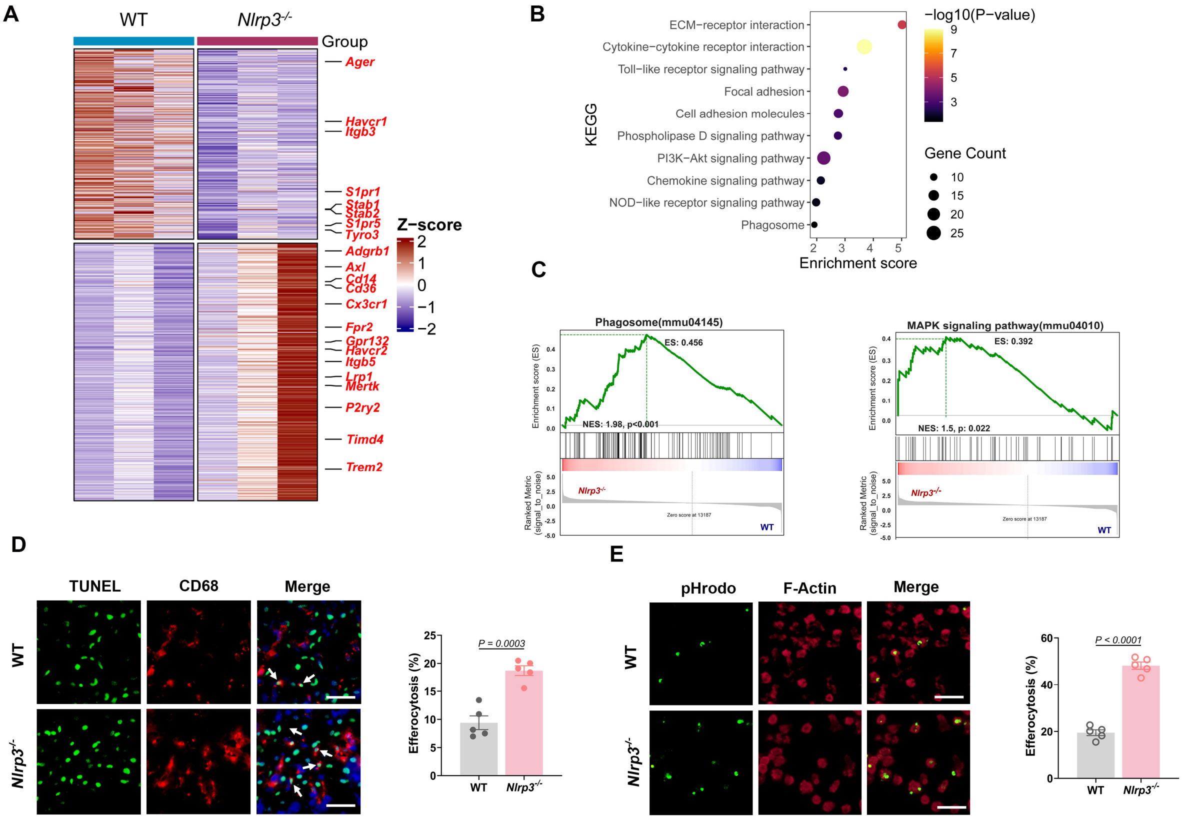

Fig. S4. *Nlrp3* Deficiency Enhances Macrophage Efferocytosis. (**A**) DEG analysis of hepatic macrophages from WT and *Nlrp3*$^{-/-}$ mice at 6 h post-reperfusion. (**B**) KEGG pathway enrichment analysis of hepatic macrophages from WT and *Nlrp3*$^{-/-}$ mice at 6 h post-reperfusion. (**C**) GSEA of hepatic macrophages from WT and *Nlrp3*$^{-/-}$ mice at 6 h post-reperfusion. (**D**) Representative co-staining images (red: CD68 [macrophage marker]; green: TUNEL [apoptotic cell marker]; blue: DAPI) and quantitative analysis of efferocytosis in liver sections from WT and *Nlrp3*$^{-/-}$ mice at 6 h post-reperfusion (scale bar = 50 μm; n=5 per group). (**E**) Representative images and quantitative analysis of F-actin (red)-labeled *Nlrp3*$^{-/-}$ BMDM engulfing pHrodo (green)-labeled apoptotic cells (scale bar = 50 μm; n=5 per group). Data was shown as Mean ± SEM.

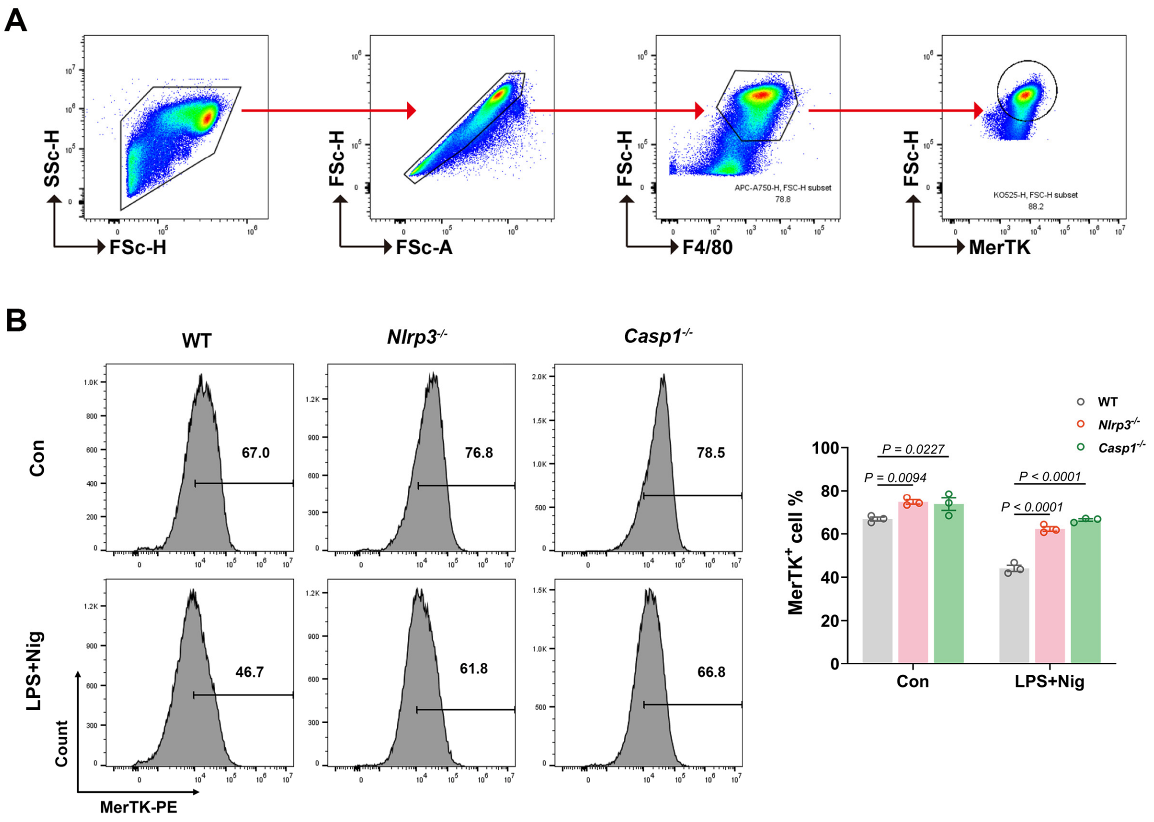

Fig. S5. ***Nlrp3/Casp1* Deficiency Increases MerTK$^+$ Cell Ratio.** BMDM were labeled with F4/80 antibody (macrophage surface marker) for flow cytometric gating. n=5 per group; Data was shown as Mean ± SEM.

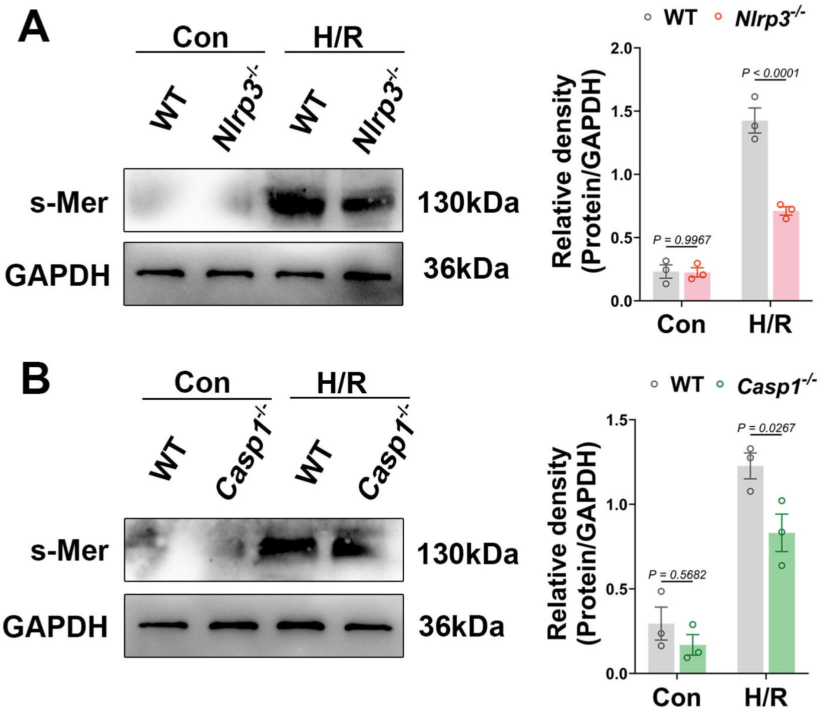

Fig. S6. Genetic deletion of *Nlrp3* or *Casp1* attenuates H/R-induced MerTK cleavage in macrophage. (**A**) Western blot analysis and quantification of s-Mer levels in culture supernatants of WT and *Nlrp3*$^{-/-}$ BMDMs under control (Con) or H/R conditions (n=3 per group). **(B)** Western blot analysis and quantification of s-Mer levels in culture supernatants of WT and *Casp1*$^{-/-}$ BMDMs under control (Con) or H/R conditions (n=3 per group). GAPDH was used as a loading control. Data was shown as mean ± SEM.

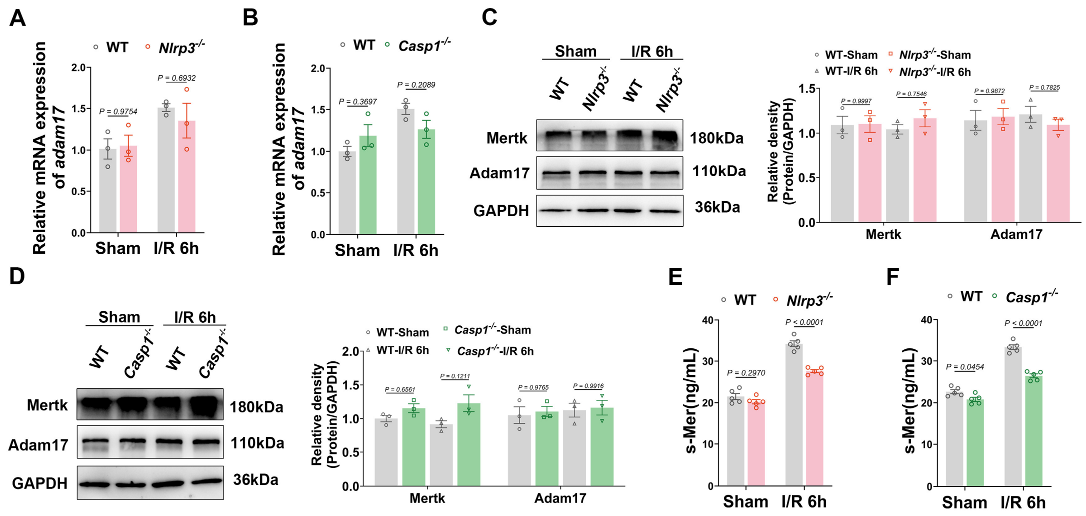

Fig. S7. *Nllrp3* or *Casp1* deficiency attenuates MerTK cleavage independent of ADAM17 expression. (**A**)qRT-PCR analysis of *Adam17* mRNA expression in livers from WT and *Nlrp3*$^{-/-}$ mice at designated time points (n=3 per group). (**B**)qRT-PCR analysis of *Adam17* mRNA expression in livers from WT and *Casp1*$^{-/-}$ mice at designated time points (n=3 per group). (**C**) Western blot analysis and quantification of Adam17 and Mertk protein levels in livers from WT and *Nlrp3*$^{-/-}$ mice at designated time points (n=3 per group). (**D**) Western blot analysis and quantification of Adam17 and Mertk protein levels in livers from WT and *Casp*$^{-/-}$ mice at designated time points (n=3 per group). (**E**) ELISA quantification of serum s-Mer levels in WT and *Nlrp3*$^{-/-}$ mice at indicated time points (n=3 per group). (**F**) ELISA quantification of serum s-Mer levels in WT and *Casp1*$^{-/-}$ mice at indicated time points (n=3 per group). Data was shown as Mean ± SEM.

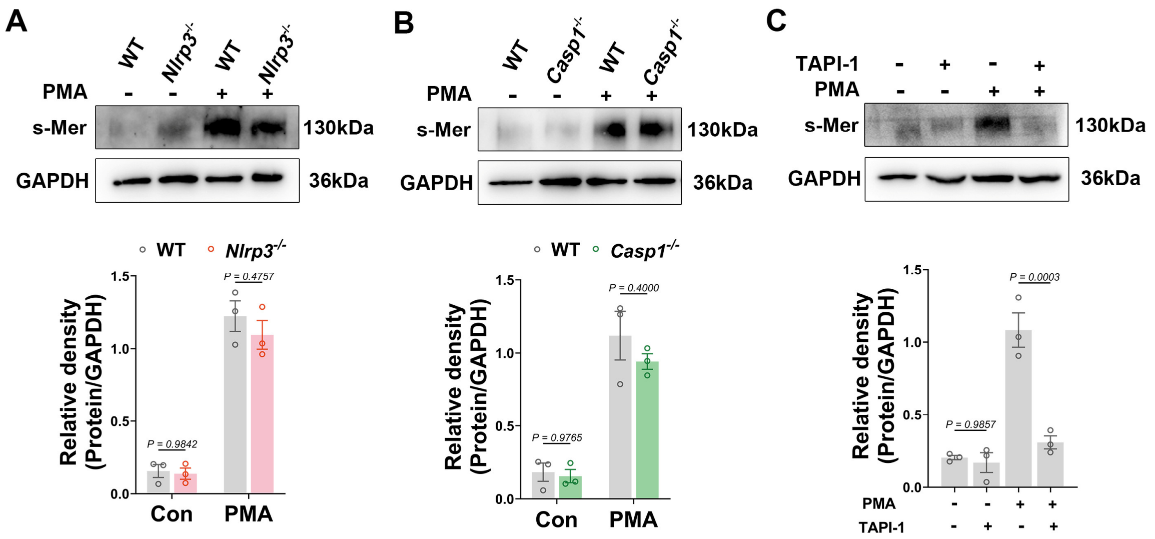

Fig. S8. *Nlrp3* or *Casp1* deficiency fails to attenuate PMA-induced MerTK cleavage in macrophages. (**A**) Western blot analysis and statistical quantification of s-Mer protein levels in WT and *Nlrp3*$^{-/-}$ BMDM treated with with or without 100 nM PMA (ADAM17 activator) for 1 hour (n=3 per group). (**B**) Western blot analysis and statistical quantification of s-Mer protein levels in WT and *Casp1*$^{-/-}$ BMDM treated with with or without 100 nM PMA (ADAM17 activator) for 1 hour (n=3 per group). (**C**) Western blot analysis and statistical quantification of s-Mer protein levels in WT BMDM treated with 100 nM PMA for 1 hours followed by 50 µM TAPI-1 (ADAM17 inhibitor) for 1 hour (n=3 per group). Data was shown as Mean ± SEM.

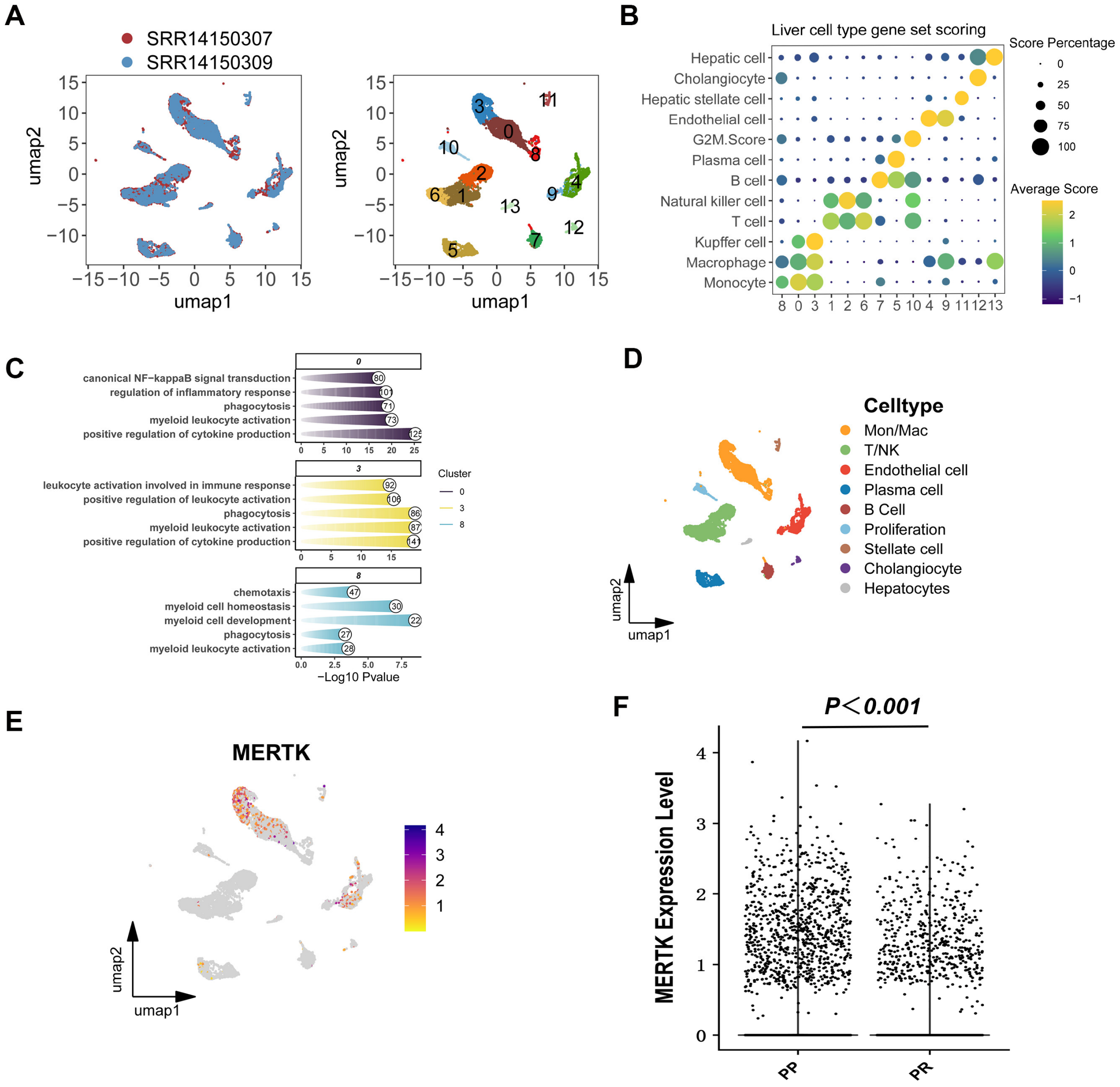

Fig. S9. scRNA-seq Analysis of Pre- and Post-Liver Transplantation Samples. (**A**) Batch correction and clustering results of single-cell transcriptomic data. (**B**) Distribution of cell type enrichment scores across clusters in liver tissue. (**C**) Functional enrichment analysis (Gene Ontology/KEGG) for clusters 0, 3, and 8. (**D**) Visualization of cell type annotations after integration. (**E**) UMAP plot showing the expression of MERTK across cell clusters, with expression levels indicated by the color scale. (**F**) Intergroup differences in MERTK gene expression across all cell types. Data was shown as Mean ± SEM.

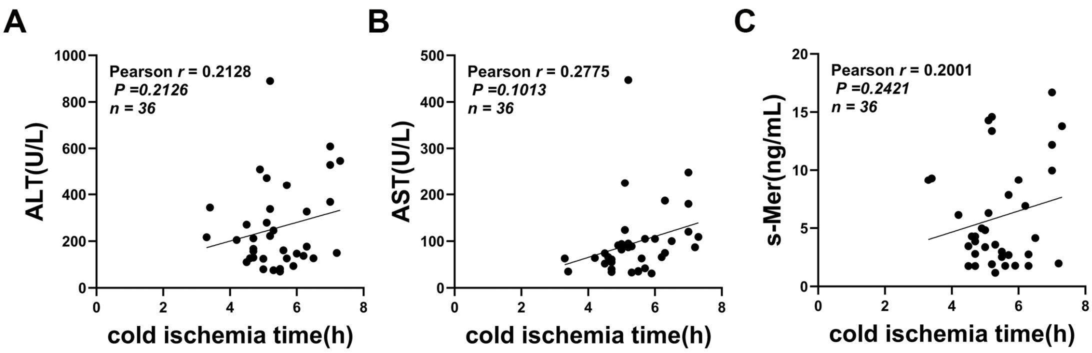

Fig. S10. No significant correlation of cold ischemia time with postoperative biomarkers in patients. Scatter plots showed the association between cold ischemia time and (**A**) ALT, (**B**) AST, and (**C**) s-Mer levels in serum samples obtained from TL patients. Data was shown as Mean ± SEM.

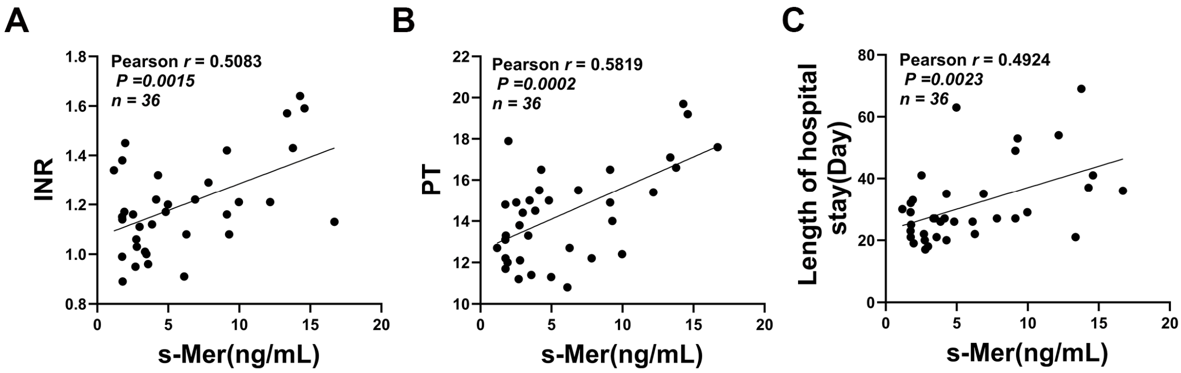

Fig. S11. Correlation analysis of serum s-Mer level and prognostic indicators in TL patients (**A**) Pearson correlation between serum s-Mer and International Normalized Ratio (INR) in TL patients at POD3 (n=36). (**B**) Pearson correlation between serum s-Mer and prothrombin time (PT) in TL patients at POD3 (n=36). (**A**) Pearson correlation between serum s-Mer and the length of hospital stay (Day) in TL patients (n=36). Data was shown as Mean ± SEM.

Table S1. Inclusion details of liver transplantation clinical samples

Type or paste caption here. Create a page break and paste in the Table above the caption.

Serial Number	Gender	Age (years)	Etiology and Diagnosis	Surgical Procedure	Donor Liver Cold Ischemia Time (h)	Steatosis Severity	Hepatic Lobular Architecture	Hepatocyte Edema	Lymphocytic Infiltration
1	Male	55	Alcoholic cirrhosis, decompensated liver function	Allogeneic liver transplantation	5.2	Mild	Normal	Mild	Mild
2	Male	56	HBV cirrhosis, decompensated liver function, hepatocellular carcinoma (HCC)	Allogeneic liver transplantation	7	Mild	Normal	Mild	Mild
3	Male	53	HBV cirrhosis, decompensated liver function	Allogeneic liver transplantation	5.1	None	Normal	Mild	Mild
4	Female	63	Autoimmune liver disease cirrhosis, decompensated liver function	Allogeneic liver transplantation	4.7	None	Normal	Mild	Mild
5	Male	46	HBV cirrhosis, decompensated liver function	Allogeneic liver transplantation	6.5	None	Normal	Mild	Mild
6	Male	63	HBV cirrhosis, decompensated liver function, HCC	Allogeneic liver transplantation	5.7	None	Normal	Mild	Mild
7	Male	53	Alcoholic cirrhosis, decompensated liver function	Allogeneic liver transplantation	5.1	None	Normal	Mild	Mild
8	Male	61	Schistosomal cirrhosis, decompensated liver function, HCC	Allogeneic liver transplantation	5	None	Normal	Mild	Mild
9	Male	54	Alcoholic cirrhosis, decompensated liver function, renal failure	Combined liver-kidney transplantation	5.5	None	Normal	Mild	Mild
10	Male	66	Alcoholic cirrhosis, decompensated liver function	Allogeneic liver transplantation	4.5	None	Normal	Mild	Mild
11	Male	54	HBV cirrhosis, decompensated liver function, HCC	Allogeneic liver transplantation	4.6	None	Normal	Mild	Mild
12	Male	50	HBV cirrhosis, decompensated liver function, HCC	Allogeneic liver transplantation	6.3	None	Normal	Mild	Mild
13	Female	63	Polycystic liver disease, decompensated liver function	Allogeneic liver transplantation	7	Moderate	Normal	Mild	Mild
14	Male	55	HBV cirrhosis, decompensated liver function, HCC	Allogeneic liver transplantation	5.3	None	Normal	Mild	Mild
15	Male	47	Alcoholic cirrhosis, decompensated liver function	Allogeneic liver transplantation	7.2	None	Normal	Mild	Mild
16	Female	48	PBC, cirrhosis with decompensated liver function	Allogeneic liver transplantation	5.5	None	Normal	Mild	Mild
17	Male	41	HBV cirrhosis, decompensated liver function	Allogeneic liver transplantation	5.6	None	Normal	Mild	Mild
18	Female	65	PBC, cirrhosis with decompensated liver function	Allogeneic liver transplantation	6.2	None	Normal	Mild	Mild
19	Male	62	HBV cirrhosis, decompensated liver function, HCC	Split liver transplantation (right hemiliver)	4.9	None	Normal	Mild	Mild
20	Male	56	HBV cirrhosis, decompensated liver function	Allogeneic liver transplantation	4.3	None	Normal	Mild	Mild
21	Male	60	Autoimmune cirrhosis, decompensated liver function	Allogeneic liver transplantation	4.7	None	Normal	Mild	Mild
22	Male	52	HBV cirrhosis, decompensated liver function	Allogeneic liver transplantation	7	None	Normal	Mild	Mild

23	Male	65	HBV cirrhosis, decompensated liver function, HCC	Allogeneic liver transplantation	5.5	None	Normal	Mild	Mild
24	Male	52	HBV cirrhosis, decompensated liver function	Allogeneic liver transplantation	5.2	Mild	Normal	Mild	Mild
25	Male	64	Alcoholic cirrhosis, decompensated liver function	Allogeneic liver transplantation	5.9	None	Normal	Mild	Mild
26	Male	54	HBV cirrhosis, decompensated liver function	Allogeneic liver transplantation	7.3	Moderate	Normal	Mild	Mild
27	Male	48	HBV cirrhosis, decompensated liver function	Allogeneic liver transplantation	6	Mild	Normal	Mild	Mild
28	Female	61	HBV cirrhosis, decompensated liver function	Allogeneic liver transplantation	5	None	Normal	Mild	Mild
29	Male	36	HBV cirrhosis, decompensated liver function, HCC	Split liver transplantation (right hemiliver)	4.7	None	Normal	Mild	Mild
30	Male	52	Alcoholic cirrhosis, decompensated liver function	Allogeneic liver transplantation	5.3	Mild	Normal	Mild	Mild
31	Male	48	HBV cirrhosis, decompensated liver function, HCC	Allogeneic liver transplantation	5.7	None	Normal	Mild	Mild
32	Male	61	HBV cirrhosis, decompensated liver function, HCC	Modified back-table technique	3.4	None	Normal	Mild	Mild
33	Male	38	HBV cirrhosis, decompensated liver function	Allogeneic liver transplantation	5.2	Mild	Normal	Mild	Mild
34	Male	45	HBV cirrhosis, decompensated liver function	Modified back-table technique	3.3	None	Normal	Mild	Mild
35	Male	53	HBV cirrhosis, decompensated liver function, HCC	Allogeneic liver transplantation	5.2	None	Normal	Mild	Mild
36	Male	46	HBV cirrhosis, decompensated liver function, HCC	Allogeneic liver transplantation	8	None	Normal	Mild	Mild

Table S2. Inclusion details of Partial hepatectomy clinical samples

Serial Number	Gender	Age (years)	Etiology and Diagnosis	Surgical Procedure	Hepatic Pedicle Clamping Time (min)	Liver Resection Extent	Steatosis Severity	Cirrhosis Grade	Tumor Size (cm)	Tumor Type
1	Male	64	Gallbladder cancer	Laparoscopic hepatectomy	30	10%	None	None	2.4×1.8	Adenoma with carcinoma in situ
2	Male	51	HBV cirrhosis HCC	Laparoscopic hepatectomy	30	15%	None	G2S2	2×2	HCC
3	Male	55	HBV cirrhosis HCC	Laparoscopic hepatectomy	60	35%	None	G2S4	10×8	HCC
4	Female	41	Steatosis, HCC	Laparoscopic hepatectomy	15	10%	Moderate	G1S1	3.5×2.5	HCC
5	Female	69	HBV cirrhosis HCC	Open hepatectomy	10	5%	None	G2S4	2×1	HCC
6	Male	52	HBV cirrhosis HCC	Laparoscopic hepatectomy	60	30%	None	G2S4	1.7×1.5	HCC
7	Female	61	HBV cirrhosis HCC	Laparoscopic hepatectomy	30	15%	None	G2S2	2.2×2.2, 1.2×1	ICC
8	Female	65	Steatosis, intrahepatic bile duct stones	Laparoscopic hepatectomy	45	30%	Mild	None	None	Low-grade intraepitheli al neoplasia
9	Male	63	HBV cirrhosis HCC	Open hepatectomy	35	20%	None	G3S4	5.5×4.5	HCC
10	Male	72	HBV cirrhosis HCC	Laparoscopic hepatectomy	25	30%	None	G2S3	2.5×2.5	HCC
11	Male	45	HBV cirrhosis HCC	Open hepatectomy	40	20%	None	G2S2	7×6	HCC
12	Male	56	HBV cirrhosis HCC	Laparoscopic hepatectomy	30	15%	None	G2S4	1.2×0.8	HCC
13	Male	66	HBV cirrhosis HCC	Laparoscopic hepatectomy	50	20%	None	G2S4	4×3.5	HCC
14	Male	53	HBV cirrhosis HCC	Laparoscopic hepatectomy	30	25%	None	G2S4	5.5×4.5	HCC
15	Male	58	Gallbladder cancer	Open hepatectomy	20	10%	Mild	None	6×2.5	Gallbladder cancer
16	Female	70	Gallbladder cancer	Open hepatectomy	16	10%	None	None	4.5×3.3	Gallbladder cancer
17	Male	59	HBV cirrhosis HCC	Laparoscopic hepatectomy	45	20%	None	G2S4	2×1.5	HCC
18	Female	67	Cholangiocarcinoma	Open hepatectomy	40	55%	None	G2S3	4×3.5	Cholangioca rcinoma
19	Female	63	Liver abscess	Open hepatectomy	45	30%	Mild	None	None	None
20	Male	64	Intrahepatic bile duct stones	Laparoscopic hepatectomy	30	30%	Mild	None	None	None
21	Male	71	HBV cirrhosis HCC	Laparoscopic hepatectomy	30	25%	None	G2S4	4×4	HCC
22	Female	70	HBV cirrhosis HCC	Laparoscopic hepatectomy	45	20%	None	G2S3	6.5×6.3	HCC
23	Female	60	Liver metastasis	Laparoscopic hepatectomy	30	25%	Mild	None	1	Fibrous nodule
24	Female	51	HBV cirrhosis HCC	Laparoscopic hepatectomy	25	15%	None	G2S4	3×2	HCC
25	Female	65	Intrahepatic bile duct stones	Laparoscopic hepatectomy	25	20%	Mild	None	None	None
26	Male	58	HBV cirrhosis HCC	Laparoscopic hepatectomy	30	25%	Mild	G2S4	7.5×7.5	HCC

Table S3. Antibody Information Table

Antibody Name	Catalog Number	Manufacturer	Applications
Anti-CD68 Rabbit pAb	GB113109-100	Servicebio	IHC, IF
Anti-Ly6G Rabbit pAb	GB11229-100	Servicebio	IHC
GAPDH Rabbit Polyclonal Antibody	ET1601-4	Huabio	WB
anti-NLRP3/NALP3 (mouse), mAb (Cryo-1)	AG-20B-0006	AdipoGen	WB, IF
anti-Caspase-1 (p20) (mouse), mAb (Casper-1)	AG-20B-0042	AdipoGen	WB, IP
Caspase-1 Rabbit pAb	A16792	Abclonal	IHC
IL1β Rabbit pAb	A16288	Abclonal	WB
IL-1 beta Mouse Monoclonal Antibody	HA601036	Huabio	WB
F4/80 Recombinant Rabbit Monoclonal Ab	HA721520	Huabio	IF
HRP Conjugated Goat anti-Mouse IgG Ab	HA1006	Huabio	WB
HRP Conjugated Goat anti-Rabbit IgG Ab	HA1001	Huabio	WB
Mouse Mer Antibody	AF591	R&D	WB
Human Mer Antibody	AF891	R&D	WB
anti-Caspase-1 (p20) (human), mAb (Bally-1)	AG-20B-0048	AdipoGen	WB
Anti-ADAM17 antibody	AB39162	Abcam	IP, IF
GM130 Antibody	DF7286	Affinity Biosciences	IF
